# Massive expression of cysteine-containing proteins causes abnormal elongation of yeast cells by perturbing the proteasome

**DOI:** 10.1093/g3journal/jkac106

**Published:** 2022-04-29

**Authors:** Shotaro Namba, Hisaaki Kato, Shuji Shigenobu, Takashi Makino, Hisao Moriya

**Affiliations:** Graduate School of Environmental and Life Sciences, Okayama University, Okayama 700-8530, Japan; Graduate School of Environmental and Life Sciences, Okayama University, Okayama 700-8530, Japan; National Institute for Basic Biology, Okazaki, 444-8585 Aichi, Japan; Graduate School of Life Sciences, Tohoku University, Sendai, Miyagi 980-8577, Japan; Graduate School of Environmental and Life Sciences, Okayama University, Okayama 700-8530, Japan

**Keywords:** yeast, fluorescent protein, cytotoxicity, protein burden, heat shock response, morphology, proteasome

## Abstract

The enhanced green fluorescent protein (EGFP) is considered to be a harmless protein because the critical expression level that causes growth defects is higher than that of other proteins. Here, we found that overexpression of EGFP, but not a glycolytic protein Gpm1, triggered the cell elongation phenotype in the budding yeast *Saccharomyces cerevisiae*. By the morphological analysis of the cell overexpressing fluorescent protein and glycolytic enzyme variants, we revealed that cysteine content was associated with the cell elongation phenotype. The abnormal cell morphology triggered by overexpression of EGFP was also observed in the fission yeast *Schizosaccharomyces pombe*. Overexpression of cysteine-containing protein was toxic, especially at high-temperature, while the toxicity could be modulated by additional protein characteristics. Investigation of protein aggregate formation, morphological abnormalities in mutants, and transcriptomic changes that occur upon overexpression of EGFP variants suggested that perturbation of the proteasome by the exposed cysteine of the overexpressed protein causes cell elongation. Overexpression of proteins with relatively low folding properties, such as EGFP, was also found to promote the formation of SHOTA (Seventy kDa Heat shock protein-containing, Overexpression-Triggered Aggregates), an intracellular aggregate that incorporates Hsp70/Ssa1, which induces a heat shock response, while it was unrelated to cell elongation. Evolutionary analysis of duplicated genes showed that cysteine toxicity may be an evolutionary bias to exclude cysteine from highly expressed proteins. The overexpression of cysteine-less moxGFP, the least toxic protein revealed in this study, would be a good model system to understand the physiological state of protein burden triggered by ultimate overexpression of harmless proteins.

## Introduction

Overexpression of proteins sometimes negatively affects cell function, but this mechanism is largely unknown. This is because the overexpression of a functional molecule, a protein, can have a variety of consequences based on its properties (Moriya 2015). While it is commonly believed that such dysfunction is caused by overexpression of cytotoxic proteins, this is not necessarily true. Even proteins that are harmless to the cell, when ultimately overexpressed, cause inhibition of proliferation by monopolizing the production resources of protein in the cell. This phenomenon is referred to as protein burden or protein cost (Dong *et al.* 1995; Snoep *et al.* 1995; Stoebel *et al.* 2008; Scott *et al.* 2010; Kafri *et al.* 2016; Eguchi *et al.* 2018; Farkas *et al.* 2018; Kintaka *et al.* 2020). Although protein burden appears to be a simple phenomenon, many details remain unclear, such as what are the main limiting molecules that cause growth inhibition and how cells cope with the abnormalities in growth caused by protein burden (Kafri *et al.* 2016, 2020; Farkas *et al.* 2018; Kintaka *et al.* 2020).

Importantly, protein burden can only be triggered by the overexpression of harmless proteins. This is because overexpression of harmful proteins causes growth inhibition at expression levels lower than the expression levels that overload the resources for protein synthesis (Moriya 2015; Eguchi *et al.* 2018; Kintaka *et al.* 2020). In other words, it is said that a protein (group) that causes protein burden is the one that can be expressed in the highest levels in the cell (and then, it causes growth inhibition). Therefore, in order to approach the phenomenon of protein burden, it is necessary to find suitable harmless proteins. Such proteins should not have any extra negative effects on cellular processes due to overexpression. They should not use extra resources in the cell other than synthesis, i.e. they should be localized in the cytoplasm and not use transport resources, they should be self-folding and not use folding resources, and they should be stable and not use resources for degradation. In addition, they must not bind nonspecifically to other proteins in the cell upon overexpression, and they must not perturb intracellular regulation. Overexpression of proteins that have characteristics producing those negative effects causes impairment of cellular functions related to their properties. Therefore, the nature of protein burden cannot be understood from the physiological state caused by the overexpression of such proteins. For example, overexpression of misfolding-prone proteins causes depletion of resources such as chaperones and proteasomes, and overexpression of transported proteins causes overloading of transporters (Geiler-Samerotte *et al.* 2011; Kintaka *et al.* 2016, 2020). This is related to the physiological conditions created by the excess of these proteins. The physiology of protein burden cannot be understood from the overexpression of these proteins, as these proteins should trigger growth inhibition at levels lower than those that cause protein burden (Moriya 2015; Kintaka *et al.* 2020).

In practice, however, it is difficult to know which proteins are harmless and suitable for investigating protein burden. In the absence of clear indicators of protein burden, the only approach to knowing which proteins are harmless enough to cause protein burden is to predict that they are likely to be harmless based on their known properties and to determine which proteins actually have the highest known expression levels that cause growth inhibition. Based on studies using the budding yeast *Saccharomyces cerevisiae* as a model for eukaryotic cells, enhanced green fluorescent protein (EGFP), the EGFP derivative yellow fluorescent protein (FP) Venus, and the red FP mCherry are considered to be such harmless proteins (Geiler-Samerotte *et al.* 2011; Kafri *et al.* 2016; Eguchi *et al.* 2018; Farkas *et al.* 2018; Kintaka *et al.* 2020). Although there are reports that these proteins can be toxic in mammalian and bacterial cells (Liu *et al.* 1999; Shemiakina *et al.* 2012; Ansari *et al.* 2016; Shen *et al.* 2017), they have no specific function in yeast and can be overexpressed in the largest quantities. They thus have been used as model proteins to analyze the cellular physiology of protein burden (Geiler-Samerotte *et al.* 2011; Kafri *et al.* 2016; Eguchi *et al.* 2018; Farkas *et al.* 2018; Kintaka *et al.* 2020). We have also found that several of the yeast glycolytic enzymes have overexpression limits equal to or greater than those of EGFP, and that these limits are determined independently of their enzymatic activity (Eguchi *et al.* 2018). In particular, Gpm1 has the highest overexpression limit among the glycolytic enzymes and may be the most appropriate candidate to investigate protein burden.

**Fig. 1.**
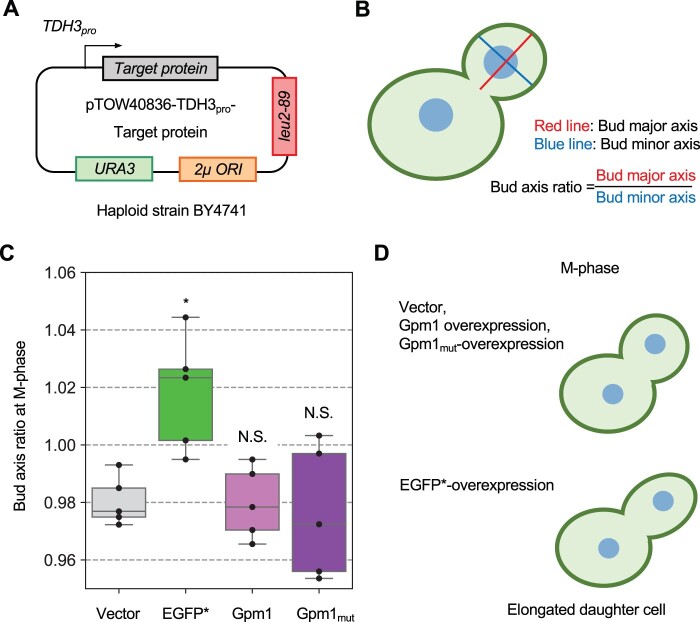
Overexpression of EGFP causes an abnormal cell elongation phenotype. a) Experimental setup of the analysis. Target proteins were expressed under the control of *TDH3* promoter (TDH3_pro_) using the multicopy plasmid pTOW40836. The haploid strain BY4741 was used as the host strain. The cells were precultured under –Ura conditions, and then cultured in YPD medium. b) Measurement of the “bud axis ratio at M phase (C114_C)” parameter using CalMorph software. The cell wall and nucleus are indicated with line and spots, respectively. c) Boxplots of the “bud axis ratio in M-phase (C114_C)” parameters for cells overexpressing the indicated proteins and the vector control. *: *P* < 0.05; NS: *P* > 0.05, likelihood ratio test; EGFP*, nonfluorescent EGFP mutant (Y66G); Gpm1mut, catalytically inactive Gpm1 mutant (H182A). d) Interpretation of the morphology of yeast cells overexpressing the indicated proteins. EGFP overexpressing strains only showed an “elongated daughter cell” phenotype.

We have recently performed a comprehensive morphological analysis of cells overexpressing EGFP, Gpm1, and the active center mutant of Gpm1 and found that only cells overexpressing EGFP exhibit an abnormal cell elongation phenotype (Kintaka *et al.* 2020) (Fig. 1). The morphology of budding yeast is a complex trait affected by a variety of factors and is a more sensitive indicator of cellular dysfunctions than mere growth inhibition (Ohya *et al.* 2005). This suggests that EGFP overexpression causes abnormal cell function that does not occur with Gpm1 overexpression.

In this study, we further analyzed the property of EGFP triggering the phenotype. By the comparison with other proteins, we found that the cysteine content was associated with the cell elongation phenotype. We further investigated the cause of cell elongation induced by overexpression of cysteine-containing proteins by analysis of aggregates, morphological analysis of mutants, and transcriptome analysis. As a result, we concluded that the perturbation of proteasomes by exposed cysteine may be the cause of cell elongation. The deleterious effect might explain the lower frequency of cysteine in more highly expressed proteins.

## Materials and methods

Strains, plasmids, assay kits, and reagents used in this study are listed in Supplementary Data 1.

### Strains, growth conditions, and yeast transformation

BY4741 was used for the CalMorph analysis (Fig. 1), and BY4743 was used for the other experiments. Cultivation and transformation of *S. cerevisiae* were performed as previously described (Amberg *et al.* 2005). Synthetic complete (SC) medium without uracil (Ura) and leucine (Leu) as indicated were used for yeast culture. Cells were cultivated at 30° otherwise noted. FY7652 was used as the fission yeast host strain. Cultivation and transformation of *Schizosaccharomyces* *pombe* were performed as previously described (Moriya *et al.* 2011). Edinburgh minimal medium (EMM) was used for yeast culture. Cells were cultivated at 30°.

### Plasmid construction

The plasmids were constructed by homologous recombination activity of yeast cells (Oldenburg 1997), and their sequences were verified by DNA sequencing.

### Genetic tug-of-war

In this study, we used the genetic tug-of-war (gTOW) method (Supplementary Fig. 1a) (Moriya *et al.*  2006, 2012), as a method to overexpress a target protein to a level that causes growth inhibition (in this study, this is referred to as “critical expression level”). In the gTOW method, the gene of the target protein is incorporated into the gTOW plasmid (here, pTOW40836) and introduced into yeast cells lacking *ura3* and *leu2* genes. The selection marker in this case is uracil (–Ura conditions). Since this plasmid has the replication origin of 2 µm plasmid (*2 µ ORI*), this plasmid becomes multicopy in the cell (about 30 in the case of the vector). When yeast cells carrying the plasmid are transferred to medium without leucine and uracil (–Leu/Ura conditions), the copy number of the plasmid in the cells increases (to about 120 in the case of the vector). This is because *leu2-89* on the plasmid is a *LEU2* allele with a large deletion in its promoter, so cells with higher plasmid copy numbers will grow faster in –Leu/Ura conditions. In other words, *leu2-89* acts as a bias to raise the plasmid copy number in –Leu/Ura conditions. If overexpression of the target protein inhibits proliferation, the plasmid copy number will be lower than the copy number that gives rise to the critical expression of that protein. In other words, the target gene acts as a bias to lower the plasmid copy number. The resulting tug-of-war between the selection biases created by the 2 genes leads to a target protein being expressed at close to the critical expression level in –Leu/Ura conditions (the green-colored area in Supplementary Fig. 1a). Based on our previous studies, FPs and glycolytic enzymes are expressed up to their critical expression levels when those proteins are expressed on the pTOW plasmid, by a strong *TDH3* promoter, and when the copy number of those plasmids is increased (Makanae *et al.* 2013; Kintaka *et al.* 2016; Eguchi *et al.* 2018). Depending on the target protein, expression under –Leu/Ura conditions is too strong for cells to proliferate. In this case, critical expression levels may be assessed in –Ura conditions (Eguchi *et al.* 2018). This is because *2 µ ORI* alone acts as a weak copy number elevation bias (Moriya *et al.* 2006). Thus, if cells harboring the gTOW plasmid incorporating the target gene show more growth retardation than the vector control, the protein is considered to be expressed at a critical expression level that causes growth inhibition. By examining these cells, we can understand the physiology of the cell when the target protein has reached its critical expression level. All “overexpression” in this study is based on the gTOW method, and it is assumed that all target proteins investigated in this study are expressed in critical amounts. Supplementary Fig. 1b shows the protein expression levels when moxGFP is overexpressed by the gTOW method as a reference example.

### Microscopic observation and morphological analysis

In the morphological analysis by CalMorph (Fig. 1), BY4741 cells overexpressing a nonfluorescent EGFP mutant (Y66G), Gpm1, or a catalytically inactive Gpm1 mutant (H182A) were cultivated until the early log phase (<5 × 10^6^ cells/ml) and fixed with formaldehyde (Fujifilm-Wako). We used nonfluorescent EGFP mutant (Y66G) (Zacharias and Tsien 2006), because its fluorescence disturbs the observation of the cell wall stained with FITC-conjugated concanavalin A. Cells were then triple-stained with FITC-conjugated concanavalin A (Sigma) for the cell wall, rhodamine-phalloidin (Thermo Fisher Scientific) for the actin cytoskeleton, and 4′,6-diamidino-2-phenylindole (Sigma) for nuclear DNA, as described previously (Ohya *et al.* 2005). Fluorescence microscopy images of the cells were acquired using an Axio Imager microscope equipped with a 6100 ECplan- Neofluar lens (Carl Zeiss, Oberkochen), a CoolSNAP HQ cooled-charge-coupled device camera (Roper Scientific Photometrics), and AxioVision software (Carl Zeiss). Micrographs of the cells were analyzed with CalMorph (ver. 1.2). The statistical analysis was performed from 5 biological replicates.

**Fig. 2.**
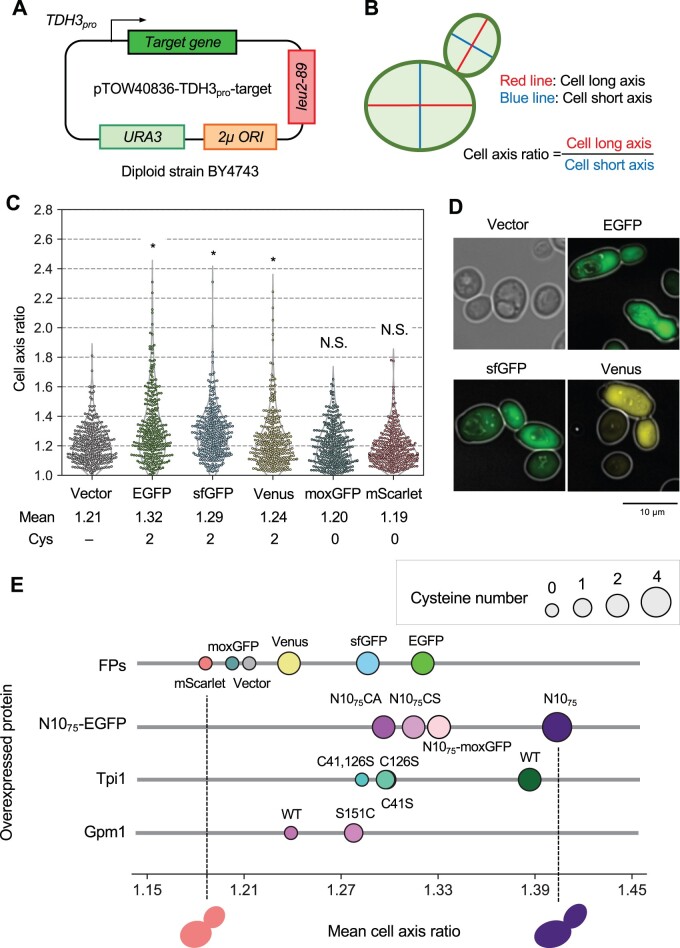
The cysteine content of overexpressed proteins is associated with the cell elongation phenotype. a) Experimental setup of the analysis. Target proteins were expressed under the control of *TDH3* promoter (TDH3_pro_) using the multicopy plasmid pTOW40836. The diploid strain BY4743 was used as the host strain. b) Measurement of the “cell axis ratio.” The round lines represent cell outlines. c) Swarm plot of cell axis ratio of cells overexpressing the indicated FPs and the vector control. Plots were based on 300 cells from 3 biological replicates. In comparison to the vector control, *: *P* < 0.05; NS: *P* > 0.05, Levene’s test with Bonferroni correction. The mean cell axis ratio (Mean) and the cysteine content (Cys) of each protein are also shown. The cells were cultured in –Leu/Ura conditions. d) Representative microscopic images of cells overexpressing EGFP, sfGFP, and Venus, as well as the vector control cells; for FP images, bright-field and pseudo-color fluorescence images were merged. e) Relationship between cysteine content and cell elongation phenotype. The mean cell axis ratio of cells overexpressing the indicated proteins is plotted. The size of the circles indicates the cysteine content of each protein. For N10_75_-GFP, except for N10_75_-moxGFP, the N10_75_ sequences were bound to EGFP. The interpreted morphology of cells with the least (mScarlet overexpression) and most (N10_75_-EGFP overexpression) elongation is also shown.

**Fig. 3.**
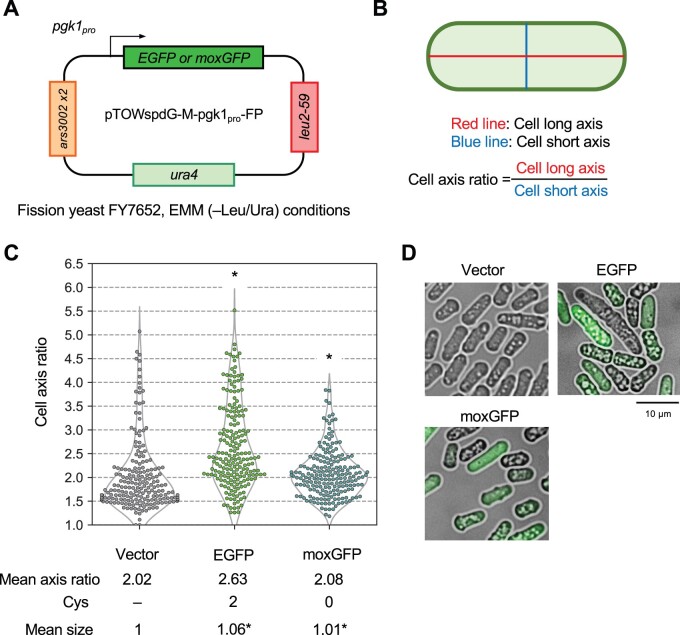
Overexpression of EGFP also causes abnormal cell growth phenotypes in the fission yeast *Schizosaccharomyces pombe*. a) Experimental setup of the analysis. The target proteins were expressed under the control of the *pgk1* promoter (pgk1_pro_) using the multicopy plasmid pTOWspdG-M. FY7652 was used as the host strain. Cells were cultured in EMM medium (−Leu/Ura). b) Measurement of the “cell axis ratio.” The rounded corner rectangle line represents the cell’s outline. c) Swarm plot of cell axis ratio of cells overexpressing EGFP and moxGFP and the vector control. Plots were based on 200 cells from 2 biological replicates. The mean cell axis ratio (Mean axis ratio), the cysteine content of each protein (Cys), and the mean cell size normalized with the vector control (Mean size) are also shown. In comparison to the vector control, *: *P* < 0.05; NS: *P* > 0.05, Levene’s test with Bonferroni correction. d) Representative microscopic images of cells overexpressing EGFP and moxGFP; for FP images, bright-field and pseudo-color fluorescence images were merged.

Cell elongation analysis in budding yeast (Fig. 2) was performed as follows (the analytical scheme was shown in Supplementary Fig. 2). BY4743 cells overexpressing a target protein were cultivated in SC–Ura or SC–Leu/Ura medium. Images of log-phase cells (OD660 = 0.8–1.0) were obtained and processed using the DMI6000 B microscope and Leica Application Suite X software (Leica Microsystems). The GFP and YFP fluorescence was observed using the GFP filter cube. Cells in the microscopic images were segmented using YeastSpotter (Lu *et al.* 2019). The morphological traits of the cells were analyzed by using CellProfiler (Ver.4.0.7) (Carpenter *et al.* 2006). For the analysis of cell elongation, we used the parameters “AreaShape_MajorAxisLength” and “AreaShape_MinorAxisLength.” We randomly chose 100 cells from 10 images for each experiment. The statistical analysis was performed on 300 cells derived from 3 biological replicates. We calculated the mean of cell axis ratio and performed Levene’s test for the statistical test. The cell elongation analysis in fission yeast (Fig. 3) was essentially the same as for budding yeast, except that the host cells were FY7652, cultured in EMM medium, and statistical analysis was performed on 200 cells derived from 2 biological replicates.

### Drug treatment

Yeast were incubated to OD660 of 1.0 and then each agent was added to the medium. Microscopic observations were performed before (0 point), 3 h after and overnight at 30°C after addition. Cycloheximide was added to a final concentration of 250 µg/ml and bortezomib to a final concentration of 100 µM/ml.

### Measuring growth rate and GFP fluorescence

Cellular growth and GFP fluorescence were measured by monitoring OD595 and Ex485 nm/Em 535 nm, respectively, every 10 min using an Infinite F200 microplate reader (Tecan). The max growth rate (MGR) was calculated as described previously (Moriya *et al.* 2006). Average values, SD, and *P*-values of Welch’s *t*-test were calculated from at least 3 biological replicates.

### Protein analysis

BY4743 cells overexpressing a target protein were cultivated in SC–Leu/Ura medium. The total protein was extracted from log-phase cells (OD660 = 0.9–1.0) with a NuPAGE LDS sample buffer (Thermo Fisher Scientific) after 0.2 mol/l NaOH treatment (Kushnirov 2000). For each analysis, the total protein extracted from 1 optical density (OD) unit of cells with OD660 was used. For total protein visualization, the extracted total protein was labeled with Ezlabel FluoroNeo (ATTO), as described in the manufacturer’s protocol, and separated by 4–12% sodium-dodecyl sulfate acrylamide gel electrophoresis (SDS-PAGE). Proteins were detected and measured using the LAS-4000 image analyzer (GE Healthcare) in SYBR–green fluorescence detection mode and Image Quant TL software (GE Healthcare). For detection of GFP, the SDS-PAGE-separated proteins were transferred to a PVDF membrane (Thermo Fisher Scientific). GFP was detected using an anti-GFP antibody (Roche), a peroxidase-conjugated secondary antibody (Nichirei Biosciences), and a chemiluminescent reagent (Thermo Fisher Scientific). The chemiluminescent image was acquired with a LAS-4000 image analyzer in chemiluminescence detection mode. The GFP and its aggregation levels were estimated as shown in Supplementary Fig. 13. We measured the GFP level in arbitrary unit (AU) as the relative intensities of GFP band within the total protein separated by SDS-PAGE, and the aggregation level in AU as the relative intensity of laddered bands above the GFP band detected by the Western blot using α-GFP antibody. Average values, SD, and *P*-values of Welch’s *t*-test were calculated from 3 biological replicates.

### High-temperature sensitivity assay

BY4743 cells overexpressing a target protein were cultivated until log-phase (OD_660_ = 0.5–0.6) in SC–Leu/Ura medium at 30°C or 38°C. Images of cells were acquired and segmented in the same manner as in “Microscopic observation and morphological analysis.” To detect dead cells, we stained cells by propidium iodide or SYTOX Green (Thermo Fisher Scientific), whose fluorescence was obtained using A (for UV) or RFP filuter cubes, respectively. To calculate the percentage of dead cells, at least 300 cells were obtained from 3 biological replicates. For yeast spot assays, BY4743 cells overexpressing a target protein were cultivated for 5 h (OD_660_ = 0.7–0.9) in SC–Ura medium at 30°C. Serial dilutions of each strain were made (OD_660_ = 0.5 × 10°–10^−3^) and plated on SC–Leu/Ura medium. Plates were incubated at 30°C or 38°C and then imaged after 5 days.

### Cell fractionation and detection of GFP

BY4743 cells with an overproduction plasmid were cultured overnight at 30°C in 25 ml SC–Leu/Ura medium. Cells were collected, cells were suspended in 1 ml of PBST [10 mM phosphate buffered saline (pH 7.4), 0.001% Tween 20] with Halt Protease Inhibitor Cocktail (Thermo Fisher Scientific). Glass beads were added to the cell suspension and the tube was vortexed 3 times for 2 min. The sample was chilled on ice for 3 min between vortexing. The cell lysate was centrifuged at 20,000 g for 10 min, and the supernatant was transferred to another tube. The precipitates were washed 5 times with 1 ml of PBST. The final precipitates were suspended in 100 ml of PBST. The sample was treated with NuPAGE sample buffer (ThermoFisher) at 70°C for 10 min, and the proteins were separated by SDS-PAGE. Total protein and GFP were detected by Ezlabel FluoroNeo and western blotting with GFP antibodies as described above.

### RNA-seq analysis

BY4743 cells overexpressing EGFP or moxGFP were cultured in SC–Leu/Ura medium and harvested at the logarithmic growth phase. RNA extraction was performed according to (Köhrer and Domdey 1991). The purified RNA was quality checked by BioAnalyzer (Agilent) or MultiNA (Shimazu), and the concentration was measured by Qubit (Thermo Fisher Scientific). Purified RNA was stored at −80°C until subsequent experiments. cDNA libraries were prepared using the TruSeq Stranded Total RNA kit (Illumina), using half the protocol of the TruSeq RNA library prep kit. The library was prepared by adding 1 µl of 142.8-fold diluted ERCC RNA Spike-in mix (Thermo Fisher Scientific) to 4 µg of total RNA. Sequencing of cDNA libraries was performed by pair-end sequencing on an illumina NextSeq 550 (Illumina). Three biological duplicates were analyzed for all strains. The sequences were checked for read quality by FastP (Chen *et al.* 2018), and then aligned using Hisat2 (Kim *et al.* 2019). The aligned data were formatted into bam files by Samtools (Li *et al.* 2009) and quantified by StringTie (Pertea *et al.* 2015). Finally, expression level variation analysis was performed by EdgeR (Robinson *et al.* 2010). For visualizing transcriptome distribution, Proteomaps (Liebermeister *et al.* 2014) was used with a custom treemap template. The raw data were deposited into Gene Expression Omnibus (accession number: GSE178244). Processed data used in Fig. 7 and Supplementary Fig. 22 is attached as Supplementary Data 2. GO enrichment analysis was performed using the Gene Lists function on the SGD website (www.yeastgenome.org/).

### Amino acid content analysis

The amino acid sequences of budding yeast 5,696 named proteins were obtained from *Saccharomyces* Genome Database (https://www.yeastgenome.org/). For protein expression levels, we used data from the literature (Kulak *et al.* 2014), which investigated protein expression levels in yeast. Highly expressed proteins were defined as those with a copy number greater than 100,000. The relative frequency of each amino acid was calculated as the actual frequency of occurrence in the proteome divided by the probability of occurrence if the proteins were randomly selected from all codons. The probability of occurrence was calculated as the number of codons encoding each amino acid divided by the number of codons excluding the termination codon [e.g. for cysteine, 2/(64–3)]. The data used in this analysis are provided in Supplementary Data 3.

### Biased differentiation of amino acid composition of genes after whole genome duplication due to their expression level

To investigate how amino acid composition and expression level of ohnologs, which are duplicated genes derived from whole genome duplication 100 million years ago, have differentiated, we obtained 548 ohnolog pairs in *S.* *cerevisiae* from the Yeast Gene Order Browser (http://wolfe.gen.tcd.ie/ygob). We downloaded their amino acid sequences from the Ensembl database (http://www.ensembl.org), and counted the number of each amino acid for each sequence. We used yeast Ribo-Seq data (Ingolia *et al.* 2009) as protein synthesis rate data. Firstly, we tested our hypothesis in which an ohnolog with higher expression levels had the lower number of cysteine in the sequence than its ohnolog partner. We classified ohnolog pairs into 2 groups, which are (Class *A*) ohnolog pairs with higher expression level and the smaller number of cysteine (Class *B*) ohnolog pairs with higher expression level and the larger number of cysteine (Fig. 8b), and investigated whether the number of (*A*) was statistically larger than that of (*B*) by exact binomial test. Note that we did not use ohnologs pairs having the same number of cysteine in their amino acid sequences. We did the same analysis for other amino acids, and showed their log_2_ ratio (*B*/*A*) in Fig. 8c. The data used in this analysis are provided in Supplementary Data 3.

## Results

### Overexpression of EGFP causes abnormal cell elongation

In this study, we used the genetic tug-of-war (gTOW) method (Moriya *et al.*  2006, 2012) (Supplementary Fig. 1), as a method to overexpress a target protein to a level that causes growth inhibition (in this study, this is referred to as “critical expression level”) (see *Materials and Methods* for more detail). We recently performed a comprehensive morphological analysis of budding yeast cells overexpressing EGFP, Gpm1 and an active center mutant of Gpm1 (Gpm1_mut_) using the CalMorph software (Ohya *et al.* 2005). These overexpressing strains were thought to be under protein burden, and commonly showed abnormal actin localization (Kintaka *et al.* 2020). Unexpectedly, however, we noticed that only the cells overexpressing EGFP exhibited a “cell elongation phenotype” (Fig. 1). Figure 1, a and b shows the plasmids for overexpression of each target protein and the analysis of one of the parameters “bud axis ratio at M-phase (C114_C)” in relation to the cell morphology of the daughter cells. As shown in Fig. 1c, significantly higher bud axis ratio at M-phase were found only in the cells overexpressing EGFP (*P < *0.05, Likelihood ratio test), but not in the cells overexpressing Gpm1 and Gpm1_mut_ cells. These results indicate that EGFP has properties that Gpm1 does not, and these properties are observed as abnormal cell elongation upon overexpression (Fig. 1d).

### Cysteine content of overexpressed proteins is associated with the cell elongation phenotype

To characterize the properties of EGFP causing cell elongation, we analyzed the morphology of cells overexpressing each of the 5 FPs with different properties (Fig. 2, a–d). While we used essentially the same plasmid as above to overexpress FPs, we used the diploid strain BY4743 instead of the haploid strain BY4741 in subsequent analyses (Fig. 2a). This is because BY4743 shows a more pronounced cell elongation phenotype than BY4741 (data not shown), probably because the diploid strains have a bipolar budding pattern (Freifelder 1960; Chant and Herskowitz 1991). We assessed the cell elongation phenotype by measuring the cell axis ratio as shown in Fig. 2b (analytical detail is shown in Supplementary Fig. 2). As shown in Fig. 2, c and d, cells overexpressing EGFP, sfGFP, and Venus contained significantly more cells undergoing cell elongation than vector controls (corrected *P < *0.05, Levene’s test), whereas no significant cell elongation was observed in strains overexpressing moxGFP and mScarlet. We note that the elongation phenotype of EGFP-overexpressing cells becomes more pronounced upon passaging (Supplementary Fig. 3).

The only difference between sfGFP and moxGFP used in this study was the cysteine content (Supplementary Fig. 4), which led us to believe that cysteine content should be a characteristic of the FP causing cell elongation. Note that the cell elongation exhibited by sfGFP-overexpressing cells is significantly milder than that of EGFP-overexpressing cells (corrected *P < *0.05, Levene’s test), suggesting that characteristics other than cysteine content are also related to cell elongation (see below).

To ascertain that cysteine content is related to the cell elongation phenotype, we next examined the effects of overexpression of EGFP with a 10-amino acid sequence containing 2 cysteines added to the N-terminus (N10_75_-EGFP; Supplementary Fig. 5a). The N10_75_ sequence was isolated from a random 10-amino acid sequence that increased the toxicity of EGFP when added to the N-terminus (Moriya 2020). As shown in Supplementary Fig. 5, b and c, cells overexpressing N10_75_-EGFP showed an even stronger cell elongation phenotype than those of EGFP. When the cysteine in the N10_75_ sequence was replaced with serine or alanine (N10_75_CS-EGFP and N10_75_CA-EGFP, Supplementary Fig. 5a), the degree of cell elongation returned to the same level as that of EGFP (Supplementary Fig. 5b). Furthermore, the addition of the N10_75_ sequence to moxGFP caused significantly more cell elongation than moxGFP (Supplementary Fig. 5, a, b, and d). These results further support the relationship between cysteine content and cell elongation.

To further confirm whether the above idea that overexpression of cysteine-containing proteins causes cell elongation is applicable to proteins other than FPs, we next analyzed the glycolytic enzymes Tpi1 and Gpm1 (Supplementary Fig. S6). Tpi1 and Gpm1 have been found to have the highest critical expression levels in yeast and are considered to be harmless proteins (Eguchi *et al.* 2018). Tpi1 contains 2 cysteines, while Gpm1 does not contain any cysteine (Supplementary Fig. 6a). We replaced 2 cysteines (C41 and C126) in Tpi1 with serines, and 1 serine (C151) in Gpm1 with a cysteine. We chose S151 in Gpm1 because the serine corresponding to this position is cysteine in a fungal ortholog (Supplementary Fig. 6b), and this substitution should have only minor effects on the function and stability of Gpm1. Overexpression of Tpi1 causes cell elongation, but substitution of cysteines mitigated this cell elongation (Supplementary Fig. 6c). In contrast, the addition of cysteine to Gpm1 exacerbated cell elongation upon overexpression (Supplementary Fig. 6d). That is, the cysteine-linked cell elongation phenotype was also seen with the overexpression of proteins other than FPs.

Figure 2e summarizes the relationship between the cysteine content of the overexpressed proteins and the cell elongation phenotype. The cysteine content of the overexpressed proteins in this study is clearly linked to the phenotype of cell elongation (*r *=* *0.80, *P = *0.00039, Supplementary Fig. 7). Note that when comparing different proteins, the cysteine content alone does not explain the degree of cell elongation. For example, overexpression of Venus with 2 cysteines causes the same degree of cell elongation as overexpression of Gpm1 without cysteines. Thus, the cell elongation phenotype would also be influenced by factors other than cysteine content. We also note that this cell elongation phenotype does not appear to be associated with an increase in cell size (Supplementary Fig. 8, a–d). The cell size of the overexpressing strains exhibiting a cell elongation phenotype is not larger than that of controls; rather, there is a weak inverse correlation between cell size and cell elongation phenotype (*r* = –0.25, Supplementary Fig. 8e). Regarding the relationship between the expression level of each FP and cell size, *r *=* *0.36 for EGFP was the maximum, but no higher correlation was found for the overexpression of any of the FPs (Supplementary Fig. 9). Thus, the reported increase in cell size due to overexpression of the red FP mCherry (Kafri *et al.* 2016) did not occur with the overexpression of these FPs in our experimental conditions.

### The cell elongation phenotype caused by overexpression of cysteine-containing EGFP is also observed in evolutionarily distant fission yeast

To ascertain whether the cell elongation phenotype caused by cysteine-containing protein is a conserved phenomenon in eukaryotic cells, we next observed cell morphology upon overexpression in fission yeast, which is evolutionarily distantly related to budding yeast. Using the gTOW plasmid for fission yeast (Moriya *et al.* 2011), we measured the cell axis ratio of fission yeast when EGFP and moxGFP were overexpressed from the *pgk1* promoter (Fig. 3, a and b). As shown in Fig. 3, c and d, only cells overexpressing cysteine-containing EGFP, but not cysteine-free moxGFP, showed more elongated cells than the vector control. Note that overexpression of moxGFP also produced a statistically significant difference in the distribution of cell populations compared with the vector control (corrected *P < *0.05, Levene’s test), but no elongated cell was observed. These results suggest that the cell elongation phenotype caused by overexpression of cysteine-containing proteins is triggered by a conserved mechanism in eukaryotic cells. In the case of fission yeast, overexpression of EGFP and moxGFP resulted in a significant cell size increase (Fig. 3c).

### Overexpression of cysteine-containing proteins is toxic, but the toxicity is modulated by additional protein properties

**Fig. 4.**
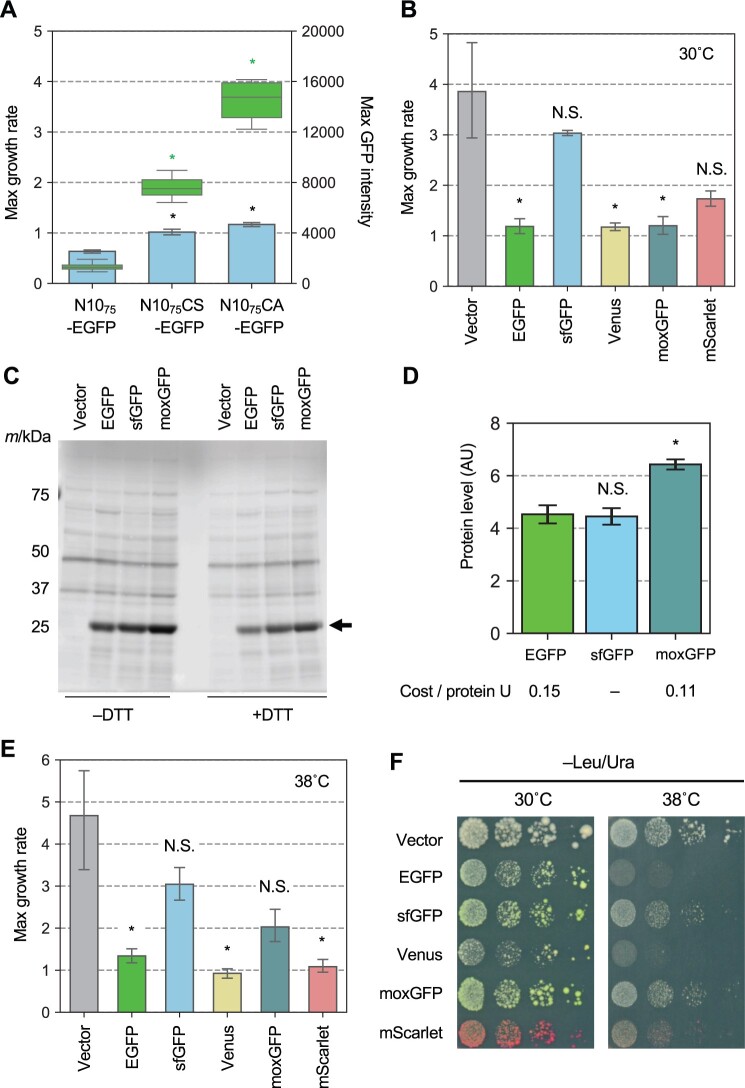
Adverse effects of overexpression of FPs on growth. a) MGR and GFP fluorescence of cells overexpressing indicated N10-EGFP. In comparison to N10_75_-EGFP-op, *: *P* < 0.05, NS: *P* > 0.05, Welch’s test with Bonferroni correction. The bars and error bars show the mean and SD of the MGRs calculated from 8 biological replicates. Box plots show GFP fluorescence obtained from the 8 biological replicates. Asterisks indicate the MGR and GFP fluorescence tests. b) MGR of cells overexpressing indicated FPs at 30°C. The bars and error bars show the mean and SD of the MGRs calculated from 8 biological replicates. In comparison to the vector control, *: *P* < 0.05; NS: *P* > 0.05, Welch’s test with Bonferroni correction. c) Gel image of SDS-PAGE-separated proteins extracted from cells overexpressing FPs. All proteins were separated after staining with a fluorescent dye. The arrow indicates the position of GFP. d) Quantification of FP expression. The intensity of the FP bands separated by SDS-PAGE as (c) was quantified, as shown in Fig. 5 and Supplementary Fig. 1a. The bars and error bars represent the mean and SD of the 3 biological replicates. In comparison to EGFP, *: *P* < 0.05; NS: *P* > 0.05, Welch’s test with Bonferroni correction. The cost per protein unit was calculated by dividing the growth rate retardation in (b) by the protein level. e) MGR of cells overexpressing indicated FPs at 38°C. The bars and error bars show the mean and SD of the MGRs calculated from 8 biological replicates. In comparison to the vector control, *: *P* < 0.05; NS: *P* > 0.05, Welch’s test with Bonferroni correction. f) Viability of cells overexpressing each FP at 30°C and 38°C. Diluted series of cells were spotted on agar medium and then cultured for 5 days. All cells in this figurine were cultured under –Leu/Ura conditions.

We next assessed whether the cysteine content of a protein is related to the cytotoxicity of the protein—the degree of growth inhibition in overexpression. We first compared the growth rates of strains overexpressing N10_75_-EGFP and its 2 cysteine substitutes shown in Supplementary Fig. 5a. This is because these proteins differ only in their cysteine content outside the folded EGFP structure, and thus their expression levels can be directly compared by measuring the fluorescence of EGFP in cells. As shown in Fig. 4a, the substitution of cysteine residues in the N10_75_ sequence for serine or alanine significantly mitigated the growth inhibition despite their increased expression (*P < *0.05, Welch’s *t*-test with Bonferroni’s multiple test correction). This result suggests that overexpression of cysteine-containing proteins is toxic.

Next, we analyzed the growth inhibition caused by the overexpression of FPs. As shown in Fig. 4b and Supplementary Fig. 10a, the growth inhibition caused by the FPs did not necessarily correspond to their cysteine content. In particular, the degree of growth inhibition of cysteine-containing EGFP and Venus and cysteine-free moxGFP was almost equally strongest, whereas cysteine-containing sfGFP did not cause significant growth inhibition. However, it should be noted that under the –Leu/Ura conditions in the gTOW method, as shown in Supplementary Fig. 1, the expression level of the target protein is autonomously adjusted to reach its critical expression level. Furthermore, critical expression levels can only be assessed when growth inhibition is observed in –Leu/Ura. In strains that do not show proliferation inhibition, the target proteins should not reach their critical expression levels. Therefore, we next measured the protein levels of EGFP, sfGFP, and moxGFP under –Leu/Ura conditions (Fig. 4, c and d). The expression level of moxGFP was about 1.4-fold higher than that of EGFP (Fig. 4d). Because overexpression of EGFP and moxGFP produced approximately the same growth inhibition (resulting in the same cost, Fig. 4b), we can say that moxGFP is about 1.4-fold less toxic than EGFP. On the other hand, the expression level of sfGFP was similar to that of EGFP under these conditions (Fig. 4d). Overexpression of sfGFP did not cause significant growth inhibition under these conditions (Fig. 4b). This indicates that while the amount of sfGFP measured here does not reach its critical expression level, the toxicity of sfGFP is at least lower than that of EGFP. At present, we do not know why sfGFP is not expressed to the level that causes growth inhibition in our experimental setup. Taken together, the cysteine-containing proteins exhibit cytotoxicity that the cysteine-less proteins do not, but which is also affected by the different properties of proteins.

Previous studies have shown that the proliferation inhibition induced by the overexpression of the 2 cysteine-containing Venus is more pronounced at higher temperatures (Farkas *et al.* 2018). We thus next investigated whether this adverse effect at high temperatures is related to cysteine content. We examined the growth of strains overexpressing 5 FPs at 38°C and found significant growth inhibition in 3 overexpressing strains, except for those overexpressing sfGFP and moxGFP (Fig. 4, e and f; Supplementary Fig. 10b). This proliferation inhibition was likely due to enhanced cell death at high temperatures in the overexpressing strains of these proteins; cells overexpressing EGFP, Venus, and mScarlet showed reduced cell viability, and gave more dead cells at 38°C (Supplementary Figs. 10 and 11). As mentioned above, although the expression levels of sfGFP probably does not reach its critical level and therefore cannot be simply compared, sfGFP did not show a strong decrease in viability at 38°C despite the inclusion of cysteine. In addition, mScarlet showed strong growth inhibition at 38°C despite the absence of cysteine. These results suggest that the cysteine content of a protein is not necessarily associated with reduced survival at high temperatures in strains overexpressing that protein. It should be mentioned here that the mechanism of growth inhibition caused by overexpression of GFP-based proteins and a red fluorescent mScarlet at high temperatures may be different. As shown in Supplementary Fig. 11, mScarlet-overexpressing strains show strong accumulation of mScarlet to the cell surface and aggregation at high temperature, whereas green FPs do not. The DsRed-based red FPs are known to form aggregates in cells and show toxicity (Shemiakina *et al.* 2012).

Importantly, cysteine-free moxGFP reaches a higher critical expression level than EGFP (Fig. 4), but still does not cause a decrease in viability at high temperatures (Supplementary Fig. 12). This implies that the inhibition of growth at high temperatures (educed viability) is caused by some specific property of EGFP, Venus, and mScarlet, and is not a general phenotype of protein burden that should occur due to the extreme overexpression of nonharmful proteins.

### Overexpression of EGFP causes protein aggregation via the S–S bond, but this may not be directly related to the cell elongation phenotype

**Fig. 5.**
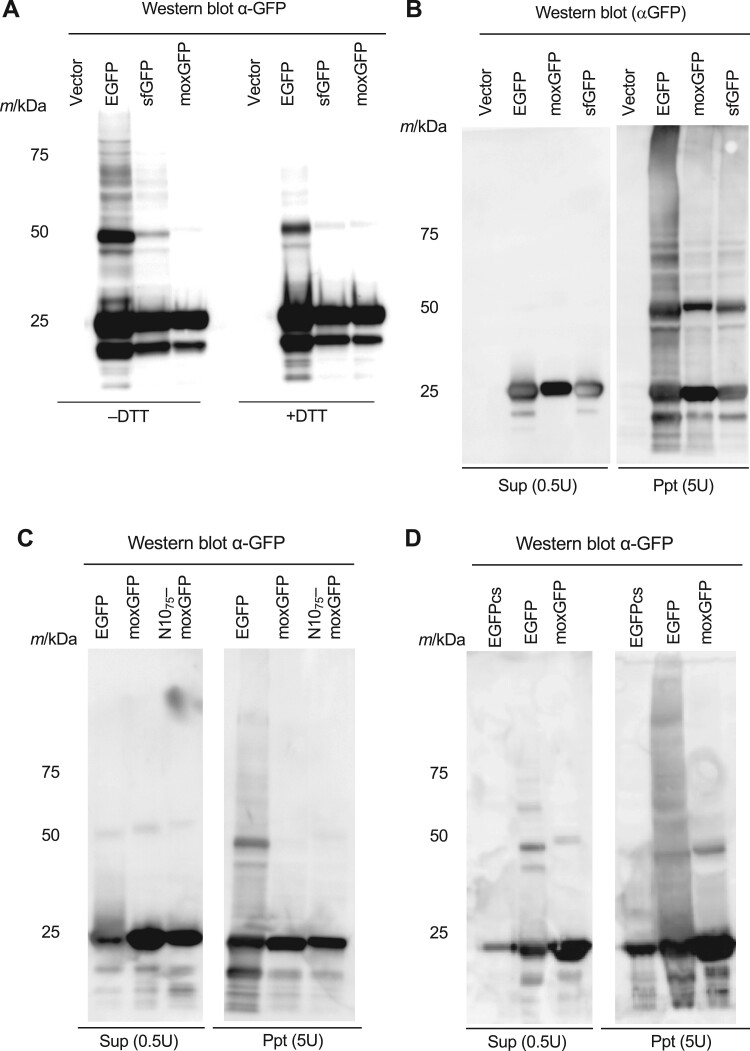
Overexpression of EGFP causes protein aggregation via the S–S bond. a) Western blotting image of proteins extracted from cells overexpressing EGFP, sfGFP, and moxGFP. Anti-GFP antibodies were used for detection. DTT-untreated (–DTT) and DTT-treated (+DTT) samples are shown. Cells cultured in –Ura were used. Western blotting images of proteins within the soluble (Sup) and insoluble (Ppt) fractions of the cells overexpressing EGFP, sfGFP, and moxGFP (c); EGFP, moxGFP, and N10_75_-moxGFP (c); EGFPcs, EGFP, and moxGFP (d). Anti-GFP antibodies were used for detection. U represents the amount of cells from which the electrophoresed protein is derived. 1U represents the amount of cells contained in 1 ml of culture showing OD_660_ of 1.0. Cells cultured in –Leu/Ura were used. The Western blotting images with the total protein images were shown in Supplementary Fig. 16.

We previously showed that overexpression of the cysteine-containing glycolytic proteins Eno2, Pgk1, and Tpi1 leads to S–S bond-mediated protein aggregation (Eguchi *et al.* 2018). It has also been reported that Tpi1 aggregates in an oxidative stress-dependent manner and that cysteine is required for this aggregation (Ford *et al*. 2019). Therefore, we hypothesized that S–S bond-linked protein aggregation might be related to the cell elongation phenotype and analyzed the intracellular properties of 3 GFP variants (EGFP, sfGFP, and moxGFP) by SDS-PAGE and western blotting. As expected, cell extracts from EGFP-overexpressing cells contained EGFP aggregation, which was seen as a high-molecular-weight ladder band (Fig. 5a; Supplementary Fig. 13b). This aggregation was lost upon treatment with the reducing agent dithiothreitol (DTT) (Fig. 5a; Supplementary Fig. 13c), suggesting that it was composed of S–S bonds. The formation of the aggregates was not lost by treatment of the cells with N-ethylmaleimide, a blocking-agent of free thiol group (Supplementary Fig. 14), suggesting that the formation of the aggregates occurred within the cells rather than during protein extraction. The level of aggregation formation was lower in the overexpressing strain of sfGFP than that of EGFP, and no aggregation was observed in the overexpressing strain of moxGFP (Fig. 5a; Supplementary Fig. 14). These results confirm that overexpression of cysteine-containing GFP causes the formation of aggregates linked by the S–S bond. Because the 2 cysteines present in EGFP and sfGFP are located within the molecule rather than on the surface of the GFP molecule (Supplementary Fig. 15), the S–S bond-mediated aggregation should be composed by GFP molecules that are not properly folded. Although sfGFP, like EGFP, contains 2 cysteines, the amount of aggregation caused by its overexpression was significantly lower than that of EGFP (Supplementary Fig. 13b, corrected *P < *0.05, Welch’s *t*-test). Thus, cysteine content is not the sole determinant of aggregation. This may be due to the fact that sfGFP folds faster and is less prone to misfolding than EGFP (Pédelacq *et al.* 2006; Eguchi *et al.* 2018). It is also possible that the amino acid substitutions that sfGFP possesses may affect the reactivity of cysteine residues, although sfGFP can also fold properly in oxidative environments (Aronson *et al.* 2011).

We next fractionated cell extracts from the overexpressing strains and examined the status of EGFP, sfGFP, and moxGFP in each fraction. We found that all of them came to the insoluble fraction, but only EGFP aggregates (higher molecular-weight bands) were found in the insoluble fraction (Fig. 5b). This indicates that these FPs misfold and insolubilize at a certain frequency upon overexpression, but the effect on subsequent aggregate formation differs depending on their cysteine contents and folding properties. To determine whether high molecular weight aggregation, such as that formed by EGFP overexpression, is associated with cell elongation, we next performed cell fractionation and Western blotting on N10_75_-moxGFP overexpressing cells. As a result, far fewer high molecular weight aggregation bands were detected (Fig. 5c). Given that overexpression of N10_75_-moxGFP causes cell elongation (Fig. 2; Supplementary Fig. 5), aggregate formation itself may not be directly related to cell elongation.

### Overexpression of EGFP, but not moxGFP triggers severe cell elongation phenotype in mutant strains

We next attempted to determine the mechanism by which overexpression of cysteine-containing proteins causes cell elongation. To this end, we observed cell morphology upon overexpression of EGFP and moxGFP for 21 mutant strains that we had identified in previous studies as having exacerbated proliferation upon EGFP overexpression (Kintaka *et al.* 2020) (Supplementary Fig. 17). Since some of those include mutant strains of *ACT1* (*act1-3*, *act1-108*, *act1-121*, and *act1-132*) and other genes involved in cell polarity (*bem1Δ*, *cdc24-1*, *exo70-29/37*, *sgt1Δ*, and *sla1Δ*), we thought that the relationship between cell polarity and cell elongation upon EGFP overexpression would also be clear.

**Fig. 6.**
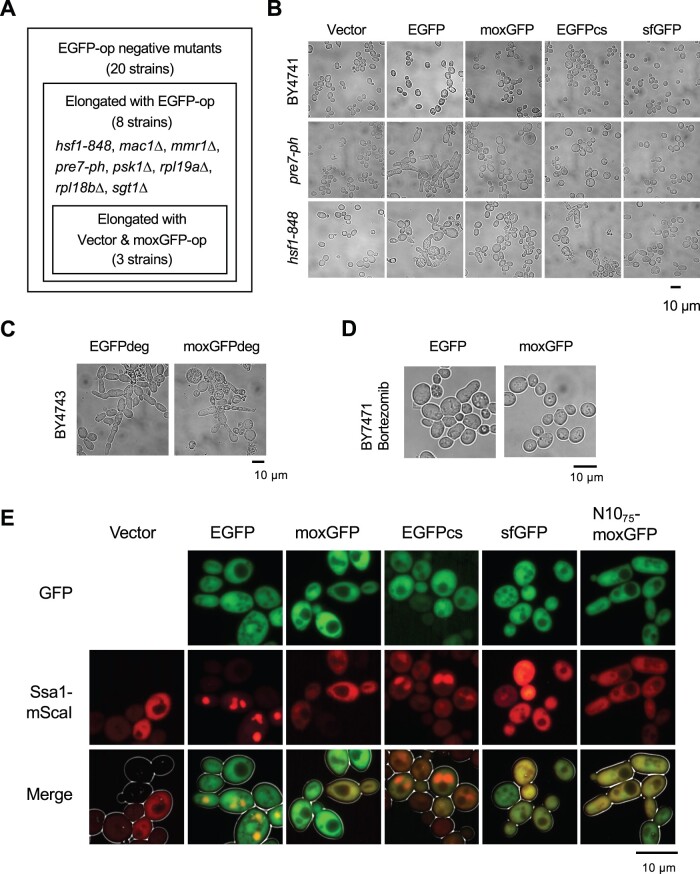
Cell biological analysis of the consequences of overexpression of GFP variants. a) Summary of morphological analysis of 21 mutants. See Supplementary Fig. 17 for images of cell morphology. “-op” means overexpression. b) Cell morphology of wild-type (BY4741), *pre7-ph*, and *hsf1-848* mutant strains upon overexpression of indicated GFP variants. c) Morphology of BY4743 cells upon overexpression of EGFPdeg and moxGFPdeg. d) Morphology of BY4741 cells upon overexpression of EGFPdeg and moxGFPdeg after the bortezomib treatment for overnight. e) Subcellular localization of Ssa1-mSca in GFP variants overexpressing strains. Ssa1-mScaI: fluorescent image of Ssa1-mScarlet-I; GFP: fluorescent image of GFP variants; Merge: superimposed image of both fluorescence and cell outlines obtained from brightfield image. The brightness and contrast of the images are adjusted so that the cells can be recognized.

This analysis resulted in 8 mutant strains that showed a strong elongation phenotype only when EGFP, but not moxGFP, was overexpressed (Fig. 6a; Supplementary Fig. 17). None of these contained mutant strains of genes involved in cell polarity, suggesting that cell elongation caused by overexpression of EGFP is not a perturbation to a group of cell polarity proteins. We note that overexpression of EGFP or moxGFP caused cell elongation in *bem1Δ* cells while overexpression of GFP, but not moxGFP, caused cell elongation in *cdc24-5* cells, suggesting that overexpression of EGFP (and moxGFP in certain cases) may cause cell elongation when certain stages of cell polarity are perturbed. On the other hand, the mutant strains contained mutations in proteins involved in multiple processes, including the heat shock response transcription factor Hsf1, proteasome component Pre7, ribosomal protein Rpl19a, and mitochondrial transport factor Mmr1. Note that overexpression of moxGFP in the *hsf1-848* mutant caused mild but significant cell elongation (Supplementary Fig. 18). These results are consistent with previous findings that the cell elongation phenotype is a complex trait involving a variety of factors (Watanabe *et al.* 2009).

### Overexpression of EGFP may cause cell elongation by perturbing proteasome function

We next focused on *PRE7*, which exhibit strong cell elongation morphology in overexpression of EGFP. Since *PRE7* encodes a proteasome, we further investigated whether perturbation of the proteasome causes cell elongation and whether this elongation is induced (only) by overexpression of EGFP. We found that (1) overexpression of EGFP, but not moxGFP, caused cell elongation in other proteasome mutations, *pre4-ph* and *pre10-ph* (Supplementary Fig. 19); (2) another proteasome mutation, *rpn12-1*, exhibited a cell elongation phenotype even in vector controls (Supplementary Fig. 19); (3) overexpression of EGFP and moxGFP with a degron sequence recognized by the proteasome (Takeuchi *et al.* 2008) caused intense cell elongation (Fig. 6c); (4) treatment with the proteasome inhibitor bortezomib caused cell elongation only with EGFP overexpression, not moxGFP (Fig. 6d; Supplementary Fig. 20). These results strongly suggest that the cell elongation phenotype induced by EGFP overexpression (at least in part) is caused by perturbation to the proteasome.

### Overexpression of EGFP leads to the formation of intracellular Hsp70 aggregates (Seventy kDa Heat shock protein-containing, Overexpression-Triggered Aggregates)

We next turned our attention to *HSF1*, whose temperature-sensitive mutation (*hsf1-848*) causes strong cell elongation upon overexpression of EGFP, but not moxGFP (Fig. 6a). In the *hsf1-848* mutant strain, overexpression of EGFP also causes strong growth inhibition (Supplementary Fig. 18, b–d). The function of Hsf1 as a transcription factor is regulated by Hsp70 (Ssa1/2 in *S. cerevisiae*) (Kmiecik *et al.* 2020). In our previous study, we found that overexpression of 3xEGFP, which consists of 3 linked EGFPs, results in the intracellular aggregates formation involving Ssa1 (Kintaka *et al.* 2020). We thus investigated whether these phenomena are related to our findings of cell morphology abnormalities and aggregate formation caused by EGFP overexpression.

We observed whether the intracellular aggregation of EGFP sequesters Ssa1 by using Ssa1-mScarlet-I (Ssa1-mSca). The results showed that Ssa1-mSca was present as intracellular aggregates in the EGFP, but not moxGFP overexpressing strain (Fig. 6e). Colocalization of Ssa1-mSca and EGFP fluorescence was not observed. This is probably due to the colocalization of misfolded, nonfluorescent EGFP with Ssa1-mSca. GFP fluorescent aggregates were seen in the 3xEGFP overexpressing strain, probably because only a portion of the 3 molecules of EGFP are misfolded (Supplementary Fig. 21a). The 3xEGFP aggregates colocalized with Ssa1-mSca aggregates (Supplementary Fig. 21a), suggesting that Ssa1-mSca colocalizes with misfolded and aggregated EGFP. The Ssa1-mSca aggregates seen in the EGFP overexpressing strain disappeared upon cycloheximide treatment of the cells (Supplementary Fig. 21, b and c). Furthermore, the fluorescence of EGFP was uniform throughout the cells (Fig. 6e), suggesting that only a part of the newly translated EGFP misfolded (and is no longer able to fluoresce), forming Ssa1-incorporated aggregates. We named this aggregate “SHOTA (Seventy kDa Heat shock protein-containing, Overexpression-Triggered Aggregates)” because, to our knowledge, such an aggregate has never been reported before. These results suggest that EGFP overexpression promotes the formation of sequestered Ssa1 structures triggered by newly synthesized and misfolded EGFP, which in turn causes dysregulation of Hsf1 activity, probably leading to growth and morphological defects in the *hsf1-848* mutant strain.

### The cause of cell elongation seemed to be related to the cysteine in the overexpressed protein, rather than misfolding and aggregation per se

Above analyses yielded *pre7-ph* and *hsf1-848* mutants in which cell elongation occurs only in overexpression of EGFP, not moxGFP. We also obtained SHOTA as a marker for intracellular aggregate formation. We thought these would be a good detection system to identify whether cysteine-containing or aggregation was responsible.

To this end, we used EGFPcs in which the 2 cysteines of EGFP were replaced by serines. Overexpression of EGFPcs did not cause the formation of insoluble high molecular weight aggregates (Supplementary Fig. 16d). This supports that the aggregates produced by overexpression of EGFPs (Fig. 5, a and b) are formed via S–S bonds. As shown in Fig. 6e, overexpression of EGFPcs caused the formation of SHOTA. Thus, SHOTA was thought to be formed independently of the cysteine of the overexpressed protein. Indeed, overexpression of both EGFP with a misfolding mutation (EGFPm3) (Kintaka *et al.* 2016) and EGFPm3cs, in which the cysteine of EGFPm3 was replaced by serine, resulted in the formation of SHOTA (Supplementary Fig. 21a). In contrast, overexpression of EGFPcs did not cause any cell elongation in both *pre7-ph* and *hsf1-848* mutants (Fig. 6b). Moreover, overexpression of N10_75_-moxGFP, which causes cell elongation even in the wild-type cells (Fig. 2; Supplementary Fig. 5), did not cause the formation of SHOTA (Fig. 6E). These results support the idea that the cause of cell elongation is related to the cysteine in the overexpressed protein, rather than misfolding and aggregation per se. Furthermore, the heat shock response triggered by sequestration of Ssa1 and activation of Hsf1 by misfolded proteins (see below) may also not directly related to cell elongation.

Note that sfGFP did not induce cell elongation even in the *pre7-ph* or *hsf1-848* mutants, despite the presence of cysteine (Fig. 6b). The folding of sfGFP is known to be robust (see above), and indeed its overexpression did not cause the formation of insoluble aggregates (Fig. 5, a and b) or SHOTA (Fig. 6e). Thus, cell elongation in these mutants may have been induced when the overexpressed protein had exposed cysteines. It is not clear at this time why overexpression of sfGFP caused significant cell elongation in the wild-type (Fig. 2), but not in the mutants. It may reflect differences in the cellular physiology caused by the mutation. Table 1 is a summary of the consequences caused by overexpression of GFP variants.

**Table 1.** Consequences caused by overexpression of GFP variants.

	EGFP	EGFPcs	sfGFP	moxGFP	N1075-moxGFP
WT elongation^#1^	+	–	±	–	+
High molecular weight aggregate^#2^	+++	–	+	–	–
*pre7-ph* elongation^#3^	+++	–	–	–	nd
*hsf1-848* elongation^#3^	+++	+	+	+	nd
Ssa1 aggregates^#3^	+	+	–	–	–

Evidence from #1: Fig. 2; #2: Fig. 5; #3: Fig. 6.

### Transcriptome analysis reveals that overexpression of EGFP and moxGFP causes different physiological states

To further investigate the physiological state of the overexpressing cells, we performed transcriptome analysis of yeast cells overexpressing EGFP and moxGFP by RNA-seq. We first visualized the RNAseq data with Proteomap (Liebermeister *et al.* 2014) to get an overall picture of the transcriptome (Supplementary Fig. 22a). We found that the overexpression strains showed decreased expression of glycolytic enzymes and increased expression of ribosomes, which seemed to be particularly pronounced in the overexpression of moxGFP.

**Fig. 7.**
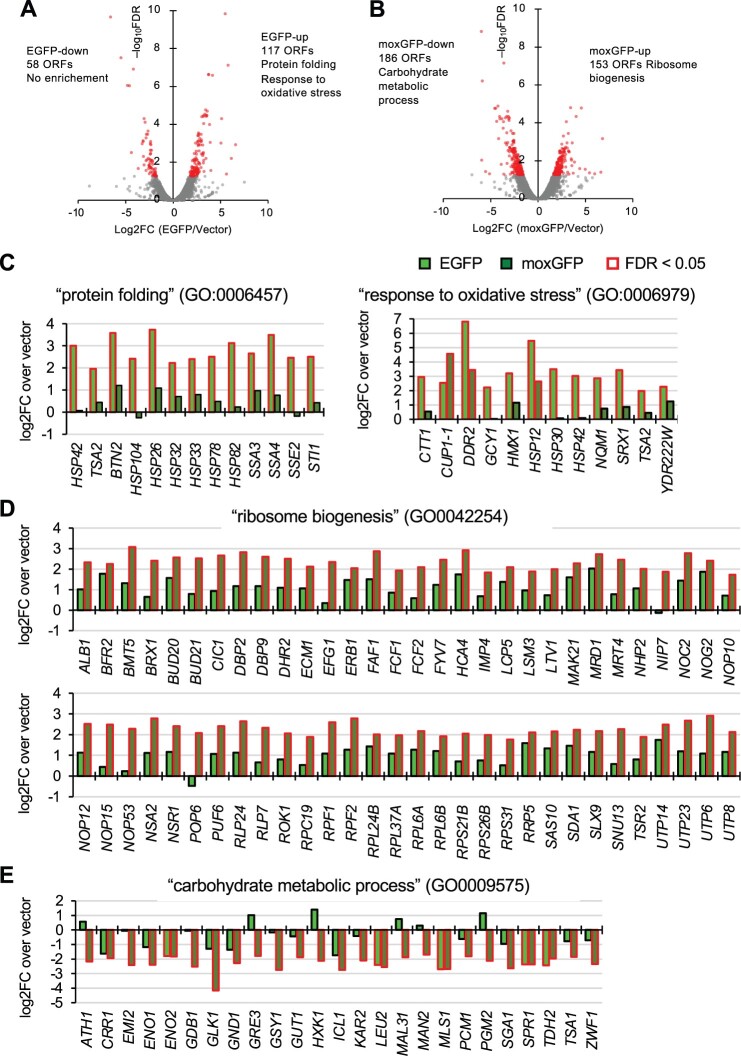
Transcriptome analysis of EGFP and moxGFP overexpressing cells obtained by RNAseq analysis. Transcriptome changes in EGFP (a) and moxGFP (b) overexpressing cells. ORFs with significant changes compared with vector control (FDR < 0.05) are highlighted. Also shown are the numbers of genes significantly up- or downregulated upon overexpression of EGFP (EGFP-up or EGFP-down) and moxGFP (moxGFP-up or moxGFP-down), and the representative GO terms enriched in those genes. c) Expression changes of EGFP-up genes linked to GO terms “protein folding (GO: 0006457)” and “response to oxidative stress (GO: 0006979).” d) Expression changes of moxGFP-up genes linked to the GO term “ribosome biogenesis (GO: 00042254).” e) Expression changes of moxGFP-down genes linked to GO terms “carbohydrate metabolic process (GO: 0009475).” All cases represent changes upon both EGFP and moxGFP overexpression. Genes with significant expression changes (FDR < 0.05) are highlighted.

Next, to analyze this more objectively, we focused our analysis on the genes whose expression was altered. Figure 7a is comparisons of RNAseq results with EGFP overexpression, moxGFP overexpression, and the vector control. Significant expression changes between these conditions (FDR < 0.05) were observed in 58–186 ORFs. We first examined changes in expression of cell cycle regulator cyclins (*CLN1-3* and *CLB1-6*), which may explain cell elongation. However, none showed significant changes (Supplementary Fig. 22b), and thus no cell cycle abnormalities could be detected, at least from this analysis. We next performed a gene ontology (GO) enrichment analysis of these gene groups. The group of genes upregulated by EGFP overexpression (EGFP-up genes) was enriched for GOs associated with protein folding and oxidative stress response (Fig. 7a and Table 2). Figure 7c shows the expression changes of EGFP-up genes linked to the 2 representative GO terms that were enriched. On the other hand, GOs associated with ribosome synthesis were enriched in the group of genes upregulated by moxGFP overexpression (moxGFP-up genes), while GOs associated with carbohydrate metabolic processes were enriched in the group of genes downregulated (moxGFP-down genes) (Fig. 7b and Table 2). Figure 7d and e shows the expression changes of moxGFP-up and moxGFP-down genes linked to the 2 representative GO terms that were enriched, respectively. Note that when comparing between EGFP and moxGFP overexpression, protein folding, oxidative stress response, and carbohydrate metabolic processes were also enriched in GOs linked to a group of genes whose expression was significantly higher in EGFP overexpressing cells (Table 2). Therefore, an important implication of this analysis is that the physiological states that occur with overexpression of EGFP and moxGFP are quite different.

**Table 2.** Representative GO terms^*a*^ significantly enriched in the differentially expressed genes of OP cells (FDR < 0.05).

GO term	*P*-value	Number of genes
EGFP > vector (117 ORFs)		
Protein folding (GO:0006457)	8.3E−05	13
Response to oxidative stress (GO:0006979)	1.3E−03	12
Vector > EGFP (58 ORFs)		
No enrichment found		
moxGFP > vector (153 ORFs)		
Ribosome biogenesis (GO:0042254)	1.1E−34	60
Vector > moxGFP (186 ORFs)		
Carbohydrate metabolic process (GO:0009575)	4.1E−05	25
moxGFP > EGFP (26 ORFs)		
No enrichment found		
EGFP > moxGFP (134 ORFs)		
Carbohydrate metabolic process (GO:0009575)	6.9E−12	28
Response to oxidative stress (GO:0006979)	1.4E−04	14
Protein folding (GO:0006457)	6.4E−04	13

a All GO analysis data are listed in Supplementary Data 2.

As noted above, overexpression of EGFP, but not moxGFP, shows a strong negative genetic interaction with the *hsf1-848* mutation, sequesters Ssa1, a repressor of transcriptional activation by Hsf1, in the aggregates (Figs. 6, b and e; Supplementary Fig. 18). This suggests that overexpression of EGFP may affect the expression of a group of genes regulated by Hsf1. In fact, many of the genes with GO “protein folding (GO: 0006457)” enriched in the EGFP-up genes contain Hsf1 targets (Solís *et al.* 2018). Therefore, we focused on the Hsf1 target and examined the transcriptional response in EGFP and moxGFP overexpressing cells. The results showed that there was indeed a significant upregulation of the Hsf1 target only in the overexpression of EGFP, but not moxGFP (Supplementary Fig. 21c and d). As noted above, overexpression of EGFP, but not moxGFP, may perturb the proteasome (Fig. 6). Indeed, transcription of *RPN4*, a regulator of the proteasome, is significantly upregulated by overexpression of EGFP (Supplementary Fig. 22b), thus perturbation of the proteasome by EGFP overexpression is also supported by the transcriptional response.

In summary, the RNAseq analyses suggest that overexpression of EGFP caused heat shock response, oxidative stress, and stress on the proteasome. Upon overexpression of moxGFP, gene expression of carbon metabolism-related proteins including a group of glycolytic enzymes was downregulated, while the expression of ribosome synthesis-related genes was upregulated.

### Cysteines are limited in the highly expressed proteins

**Fig. 8.**
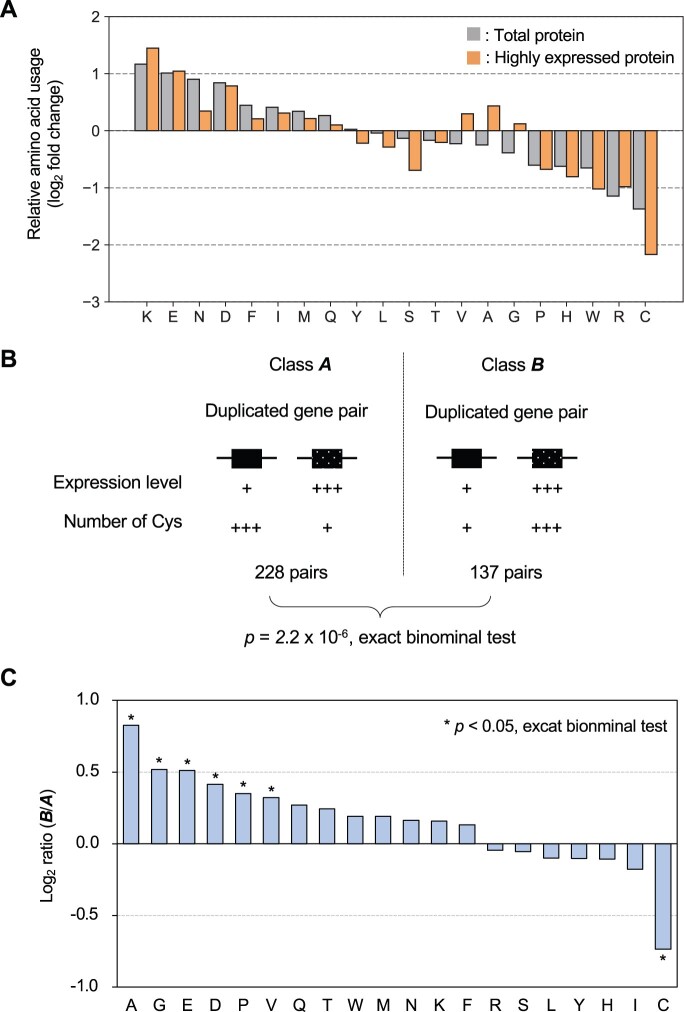
Cysteines are excluded from highly expressed proteins. a) Relative usage of each amino acid in the yeast proteome. The figure shows the comparison between the expected frequency of use from the codon table and the actual frequency of use in the proteome. Total protein: total protein of yeast, highly expressed protein: protein with more than 100,000 molecules per cell (Kulak *et al.* 2014). b) Relationship between cysteine content and expression level in proteins encoded by duplicated genes (ohnologs). Two classes of duplicated gene pairs according to the relationship between expression levels and cysteine levels. Class *A* pairs have a negative relationship between expression level and cysteine content, while Class *B* pairs have a positive relationship between expression level and cysteine content. Duplicated gene pairs in yeast are significantly more likely to belong to Class *A*. c) The ratio of Class *B* pairs to Class *A* pairs when the same analysis as in (b) is performed for each amino acid.

The results so far suggest that high expression of cysteine-containing proteins has a negative impact on cell function. In fact, the relative usage of cysteine in proteins is the lowest among all amino acids, and the trend is stronger for proteins with high expression levels (Fig. 8a). This fact suggests that cysteine has an evolutionary tendency to be excluded from proteins with high expression levels. Therefore, we next performed a more detailed evolutionary analysis of this tendency.

The budding yeast *S. cerevisiae* experienced a whole genome duplication about 100 million years ago and has duplicated gene pairs (ohnologs) in its genome (Wolfe 2015). If cysteine is detrimental to high protein expression, one would expect that the more highly expressed duplicated genes would tend to have lower cysteine content. Indeed, of the 365 differentially expressed duplicated gene pairs, 228 of higher expressed duplicated pairs had lower cysteine content, which was statistically significant (Fig. 8b). A similar analysis was performed for all amino acids, and only cysteine showed this trend (Fig. 8c). The results of these evolutionary analyses strongly indicate that high expression of cysteine-containing proteins has a negative impact on cell function.

## Discussion

**Fig. 9.**
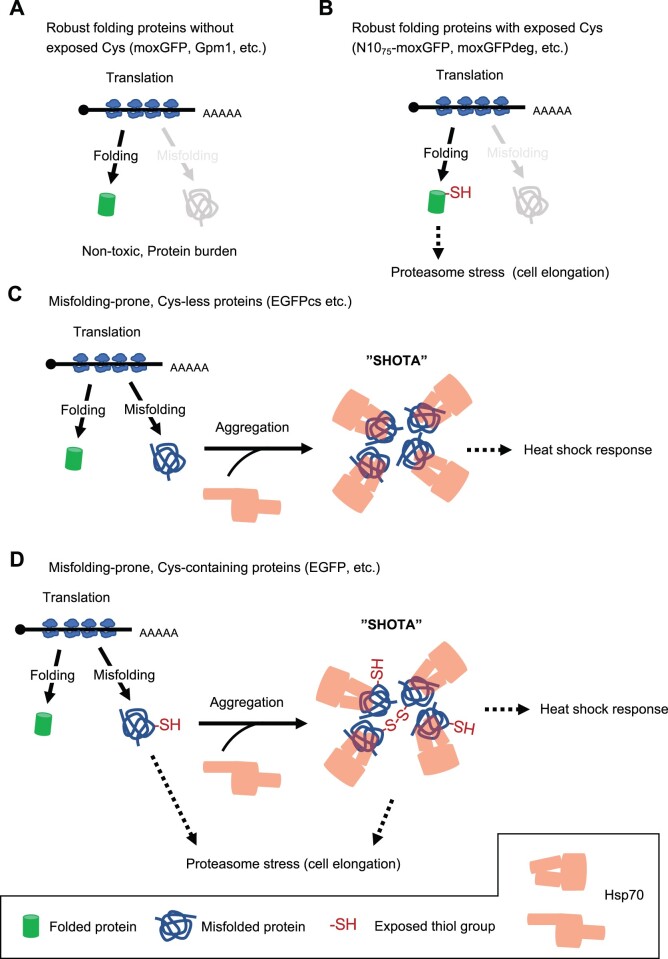
Model diagram summarizing the findings of this study. Details are provided in the body of *Discussion*.

In this study, we have shown evidence that extreme overexpression of cysteine-containing proteins results in abnormal cell elongation and proliferation in yeast. Based on the experimental data we presented in this study, we propose a model shown in Fig. 9 as the mechanism responsible for this. In this model, exposed cysteine perturbs the proteasome, which in turn causes cell elongation as follows. (1) Overexpression of cysteine-free proteins such as moxGFP and Gpm1 does not perturb the processes responsible for cell elongation (evidence shown in Figs. 1, 2, and 9a). In addition, proteins with robust folding properties, such as sfGFP, do not strongly perturb the processes responsible for cell elongation because the cysteines inside the protein are not exposed (evidence shown in Figs. 2 and 6); (2) even for proteins that fold robustly and do not aggregate, when cysteines are exposed, their overexpression perturbs the proteasome and causes cell elongation. These proteins include N10_75_-moxGFP and moxGFPdeg (evidence shown in Figs. 2, 5, 6, and 9b). (3） Overexpression of proteins with low folding properties but no cysteine, such as EGFPcs, forms Hsp70/Ssa1 aggregates (SHOTA) in the cell due to misfolding during the translation. This presumably causes a heat shock response, but not cell elongation (evidence shown in Figs. 6 and 9c). (4) Overexpression of cysteine-containing proteins with low folding properties, such as EGFP, results in misfolded, exposed cysteine. This perturbs the proteasome and causes cell elongation. Such proteins also cause the formation of SHOTA, which induces a heat shock response that is probably unrelated to cell elongation (evidence shown in Figs. 2, 6, 7, and 9d). Overexpression of such proteins also forms insoluble aggregates with large molecular weight via S–S bonds (evidence shown in Fig. 5), while it is unclear whether this is related to cell elongation.

Note that there are still some points in this model (and our study) that have not been clarified. First, there are questions about how cysteine-containing proteins perturb the proteasome and how this perturbation leads to cell elongation. With respect to the former, we consider the possibility that cysteine is involved in substrate recognition by proteasomes. Indeed, cysteine is known to be essential for the recognition of cODC degron by the proteasome (Takeuchi *et al.* 2008). If proteins with exposed cysteine are overexpressed, they may bind to the proteasome and compete with other substrates. With respect to the latter, this may be due to delayed degradation of Cln2, one of the substrates of the proteasome. It has been reported that inhibition of Cln2 degradation (by the proteasome) causes cell elongation (Schweitzer *et al.* 2005). Second, this perturbation to the proteasome explains only part of the cell elongation caused by EGFP overexpression. As shown in Fig. 6 and Supplementary Fig. 17, overexpression of EGFP also causes cell elongation in nonproteasome mutants. It is possible that the proteasome is also perturbed in these mutant strains, but there is currently no direct evidence.

We found that overexpression of EGFP promotes the formation of aggregates via S–S bonds (Fig. 5). Although it has been reported that S–S bond-mediated aggregate formation of FPs occurs in the endoplasmic reticulum, an oxidative environment (Costantini *et al*. 2015), it is not obvious that S–S bonds are formed in the cytosolic environment, which is generally believed to be reducing. On the other hand, as shown in Fig. 7c, transcription of oxidative stress response genes was significantly elevated in cells overexpressing EGFP. This suggests that overexpression of EGFP itself may disrupt the cytoplasmic redox state.

In addition to transcription and translation, proteins functioning in the cell are processed by various processes such as transport, post-translational modification, and degradation. Each of these processes must have different capacities depending on the amount of resources allocated to it. Overexpression of proteins to be treated by each process leads to cell dysfunction because the capacity of those processes becomes excessive and the proteins that should be treated by those processes are not able to be treated. We call such a condition “resource overload” (Moriya 2015; Kafri *et al.* 2016; Kintaka *et al.* 2020). The process of protein synthesis, where practically all proteins are processed, is considered to have the largest capacity among the various processes (Moriya 2015; Kafri *et al.* 2016, 2020; Eguchi *et al.* 2018; Kintaka *et al.* 2020). The state in which the capacity (and only the capacity) of the synthesis process is overloaded is called “protein burden,” among other resource overloads (Moriya 2015; Kafri *et al.* 2016; Eguchi *et al.* 2018; Kintaka *et al.* 2020). Protein burden is thought to cause a depletion of transcriptional and translational resources (Moriya 2015; Kafri *et al.* 2016; Eguchi *et al.* 2018; Kintaka *et al.* 2020), but its physiological state and cellular response are not well understood. By definition, protein burden is a situation in which a protein that does not overload any process other than synthesis is overexpressed and causes growth inhibition. However, it is not easy to know what kind of protein “does not overload processes other than synthesis” in the first place. Such a protein would be one that folds on its own (without using chaperone resources), does not localize (without using localization resources), is not actively degraded (without using degradation resources), has no proteins to interact with, and does not disrupt physiological functions when in excess. Several researchers have used specifically modified FPs as such examples (Kafri *et al.* 2016; Farkas *et al.* 2018; Kintaka *et al.* 2020). Farkas et al. (2018) reported that an excess of Venus, a yellow fluorescent protein, disrupts proteostasis involving intracellular chaperones. We have also found that 3xEGFP adversely affects the proteasome (Kintaka *et al.* 2020). These examples, however, contradict the definition of protein burden in the first place. That is, they are proteins that overload more limited resources other than protein synthesis, and thus are not appropriate proteins to investigate protein burden. In fact, in this study, we found that EGFP and Venus, probably because of their cysteine content, cause abnormal cell elongation and sensitivity to high temperature in their overexpressing strains; EGFP also aggregates intracellularly, engulfing and depleting Hsp70 in its aggregates and causing Hsf1-mediated heat shock response. Importantly, an excess of moxGFP, which does not contain cysteine (and has higher folding properties), did not induce such a phenotype, but showed higher expression limits than EGFP. In other words, moxGFP seems to be the most appropriate model protein to investigate protein burden at this time.

Interestingly, in cells overexpressing moxGFP, transcription of ribosomal proteins and proteins involved in ribosome synthesis was enhanced (Fig. 7d). This may be a true cellular response to the depletion of synthesis resources, or protein burden, caused by the massive production of harmless proteins. As mentioned above, it is recognized that the nature of protein burden is growth inhibition which occurs when protein synthesis resources are monopolized and depleted for the synthesis of harmless proteins, and the synthesis of other proteins essential for growth is reduced due to competition. However, it is not yet clear what exactly are the limiting factors that are depleted during protein burden, or the proteins that “lose out to competition” to cause growth inhibition. Based on the transcriptional response of moxGFP overexpression, we suspect that the latter may be ribosomal proteins. Our current hypothesis is that protein burden decreases synthesis, possibly translation of ribosomal proteins, and to counteract this, the cell increases transcription of ribosomes. As far as we know, this is the first time that such a response has been observed in eukaryotic cells, and a detailed analysis of the mechanism is awaited.

In this study, we showed that the (artificial) overexpression of cysteine-containing proteins has adverse effects on cell function. This negative effect of cysteine probably acts as an evolutionary bias to exclude cysteine from highly expressed proteins (Fig. 7). Cysteine is an important amino acid that contributes to the structural stabilization of proteins, the activity of enzymes, and metal binding in proteins. On the other hand, the thiol group of cysteine is highly reactive and susceptible to the redox state in the cell. High concentrations of cysteine-containing proteins can cause unwanted intermolecular and intramolecular interactions that can interfere with the function of important proteins or form cytotoxic aggregates that can adversely affect cell function. It is believed that there are evolutionary principles that avoid unwanted interactions and misfolding, especially in highly expressed proteins (Drummond *et al.* 2008; Yang *et al.* 2012). The inclusion of cysteine is a prime example in support of these hypotheses.

## Data availability

Strains and plasmids are available upon request. The authors affirm that all data necessary for confirming the conclusions of the article are present within the article, figures, and tables. Gene expression data are available at Gene Expression Omnibus (accession number: GSE178244).

Supplemental material is available at *G3* online.

## Supplementary Material

jkac106_Data_S1

jkac106_Data_S2

jkac106_Data_S3

jkac106_Supplemental_Material_Legends

jkac106_Supplemental_Figures
