# Supplementary material for: Massive expression of cysteine-containing proteins causes abnormal elongation of yeast cells by perturbing the proteasome
Source: G3 (Bethesda). 2022 Apr 29;12(6):jkac106. doi: 10.1093/g3journal/jkac106 (PMC9157148; doi:10.1093/g3journal/jkac106)
Supplement: jkac106_Supplemental_Material_Legends [file jkac106_supplemental_material_legends.docx]

**Supplementary materials**

**Supplementary figures** (provided in Supplementary_Figure.docx)

- Figure S1. Genetic tug-of-war (gTOW) method used for overexpression in this experiment.
- Figure S2. Analytical scheme of yeast cell morphology.
- Figure S3. EGFP-overexpressing cells elongate more markedly with continued passaging.
- Figure S4. Amino acid sequence alignment of EGFP, sfGFP, and moxGFP.
- Figure S5. The addition of cysteines to EGFP exacerbates the cell elongation phenotype
- Figure S6. Cysteine residues in the glycolytic enzymes Tpi1 and Gpm1 are associated with the cell elongation phenotype caused by their overexpression.
- Figure S7. Relationship between the cysteine content of the proteins investigated in this study and the cell elongation phenotype (mean cell axis ratio) when they are overexpressed.
- Figure S8. Size of cells overexpressing proteins.
- Figure S9. Scatterplots for cell size and fluorescence intensity.
- Figure S10. Growth curve of cells overexpressing fluorescent proteins.
- Figure S11. Microscopic images of cells overexpressing fluorescent proteins cultured at 38°C.
- Figure S12. Percentage of dead cells in a cell population overexpressing fluorescent proteins.
- Figure S13. Quantification of overexpressed proteins and aggregation bands.
- Figure S14. Evidence that disulfide bonding of EGFP occurs intracellularly, not during protein extraction.
- Figure S15. The thiol groups of the two cysteines of EGFP are located inside the structure.
- Figure S16. Overexpression of EGFP causes protein aggregation via the S-S bond.
- Figure S17. Microscopic images of cells overexpressing EGFP and moxGFP, as well as the vector control cells.
- Figure S18. Effects of EGFP and moxGFP overexpression on the hsf1-848 mutation
- Figure S19. Microscopic images of mutant cells overexpressing EGFP and moxGFP, as well as the vector control cells.
- Figure S20. Bortezomib treatment of overexpressing cells
- Figure S21. Behavior of Ssa1 aggregates in overexpressing cells
- Figure S22. Transcriptome analysis of EGFP and moxGFP overexpressing cells

**Supplementary data files**

- Data S1. Key_Resource_Table.xlsx
- Data S2. RNAseq_data.xlsx
- Data S3. AA_compositon_analysis.xlsx
