# Supplementary material for: Massive expression of cysteine-containing proteins causes abnormal elongation of yeast cells by perturbing the proteasome
Source: G3 (Bethesda). 2022 Apr 29;12(6):jkac106. doi: 10.1093/g3journal/jkac106 (PMC9157148; doi:10.1093/g3journal/jkac106)
Supplement: jkac106_Supplemental_Figures [file jkac106_supplemental_figures.docx]

**Supplementary Information for**

Massive expression of cysteine-containing proteins causes abnormal elongation of yeast cells by perturbing the proteasome

Shotaro Namba^1^, Hisaaki Kato^1^, Shuji Shigenobu^2^, Takashi Makino^3^, Hisao Moriya^1,*^

1. Graduate school of environmental and life sciences, Okayama University

2. National Institute for Basic Biology

3. Graduate School of Life Sciences, Tohoku University

*Correspondence to hisaom@okayama-u.ac.jp

[Figure S1. Genetic tug-of-war (gTOW) method used for overexpression in this experiment. 3](#_Toc99371581)

[Figure S2. Analytical scheme of yeast cell morphology. 5](#_Toc99371582)

[Figure S3. EGFP-overexpressing cells elongate more markedly with continued passaging. 6](#_Toc99371583)

[Figure S4. Amino acid sequence alignment of EGFP, sfGFP, and moxGFP. 7](#_Toc99371584)

[Figure S5. The addition of cysteines to EGFP exacerbates the cell elongation phenotype 8](#_Toc99371585)

[Figure S6. Cysteine residues in the glycolytic enzymes Tpi1 and Gpm1 are associated with the cell elongation phenotype caused by their overexpression. 11](#_Toc99371586)

[Figure S7. Relationship between the cysteine content of the proteins investigated in this study and the cell elongation phenotype (mean cell axis ratio) when they are overexpressed. 12](#_Toc99371587)

[Figure S8. Size of cells overexpressing proteins. 14](#_Toc99371588)

[Figure S9. Scatterplots for cell size and fluorescence intensity. 16](#_Toc99371589)

[Figure S10. Growth curve of cells overexpressing fluorescent proteins. 17](#_Toc99371590)

[Figure S11. Microscopic images of cells overexpressing fluorescent proteins cultured at 38°C. 19](#_Toc99371591)

[Figure S12. Percentage of dead cells in a cell population overexpressing fluorescent proteins. 20](#_Toc99371592)

[Figure S13. Quantification of overexpressed proteins and aggregation bands. 21](#_Toc99371593)

[Figure S14. Evidence that disulfide bonding of EGFP occurs intracellularly, not during protein extraction. 22](#_Toc99371594)

[Figure S15. The thiol groups of the two cysteines of EGFP are located inside the structure. 23](#_Toc99371595)

[Figure S16. Overexpression of EGFP causes protein aggregation via the S-S bond. 25](#_Toc99371596)

[Figure S17. Microscopic images of cells overexpressing EGFP and moxGFP, as well as the vector control cells. 27](#_Toc99371597)

[Figure S18. Effects of EGFP and moxGFP overexpression on the hsf1-848 mutation 27](#_Toc99371598)

[Figure S19. Microscopic images of mutant cells overexpressing EGFP and moxGFP, as well as the vector control cells. 28](#_Toc99371600)

[Figure S20. Bortezomib treatment of overexpressing cells 29](#_Toc99371601)

[Figure S21. Behavior of Ssa1 aggregates in overexpressing cells 30](#_Toc99371602)

[Figure S22. Transcriptome analysis of EGFP and moxGFP overexpressing cells 33](#_Toc99371603)

**
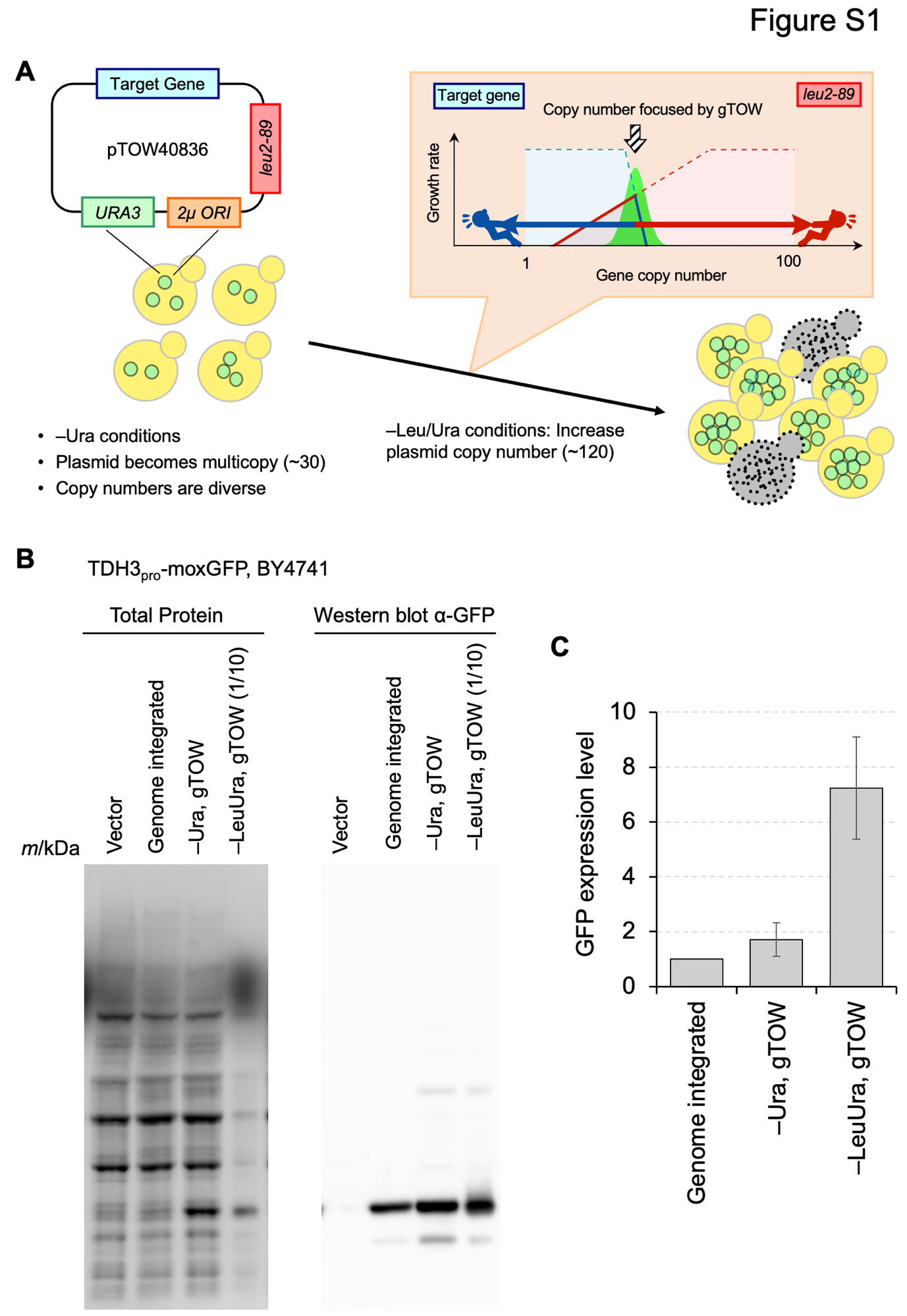
**

Figure S1. Genetic tug-of-war (gTOW) method used for overexpression in this experiment. (**A**) The gTOW method. Detailed explanations are given in Materials and Methods. (**B**) Western blotting and the total protein images of proteins extracted from cells overexpressing moxGFP from a genome-integrated TDH3_pro_-moxGFP (to the *FCY1* locus), TDH3_pro_-moxGFP on the gTOW plasmid under –Ura or –Leu/Ura conditions. Anti-GFP antibodies were used for detection. (**C**) Quantification of moxGFP detected by the Western blotting in **B**. The density of moxGFP bands in Western blotting was quantified and normalized by Genome integrated; the mean and standard deviation (error bars) of three measurements are shown.

**
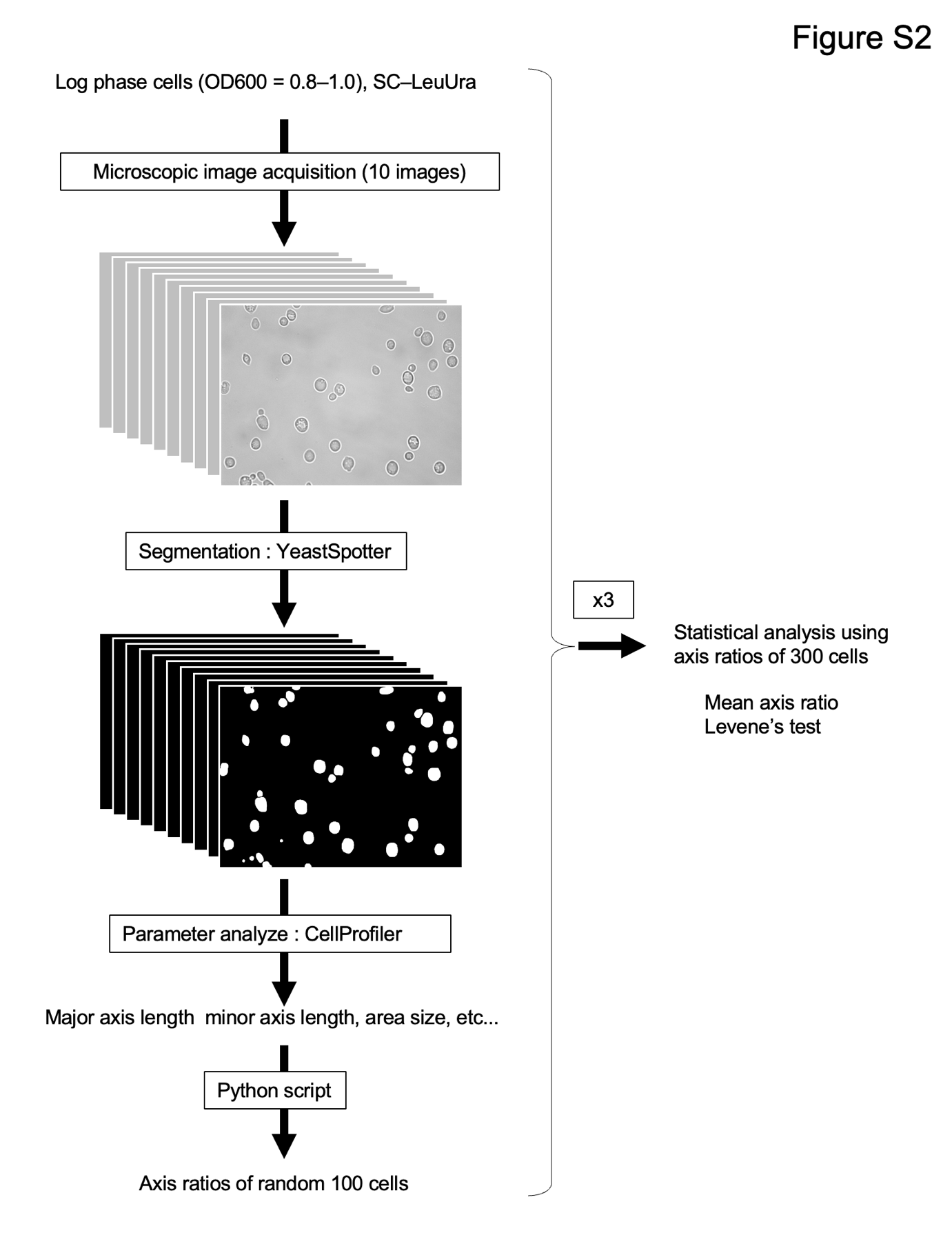
**

Figure S2. Analytical scheme of yeast cell morphology. The details of the analysis scheme are described in Materials and Methods.

**
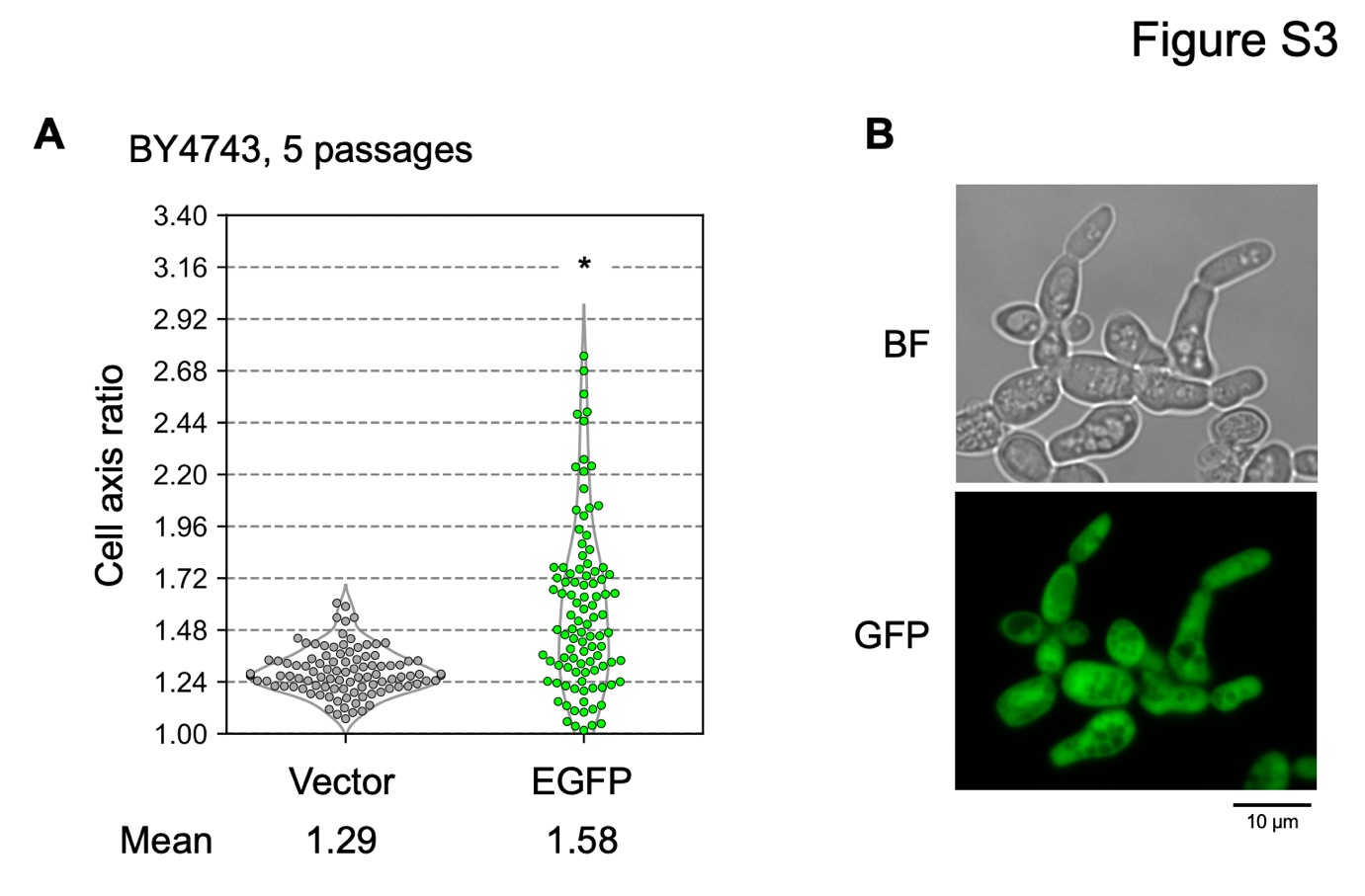
**

Figure S3. EGFP-overexpressing cells elongate more markedly with continued passaging. The cells were successively cultured in SC–Leu/Ura medium for five days with one passage per day. The cell morphology analysis was performed using randomly selected 100 cells (n = 1) and shown with swarm plots (**A**). *; *p* = 3.5E–14, Levene’s t-test. (**B**) Representative images of EGFP-overexpressing cells, showing no accumulation of EGFP at specific sites in the cells.


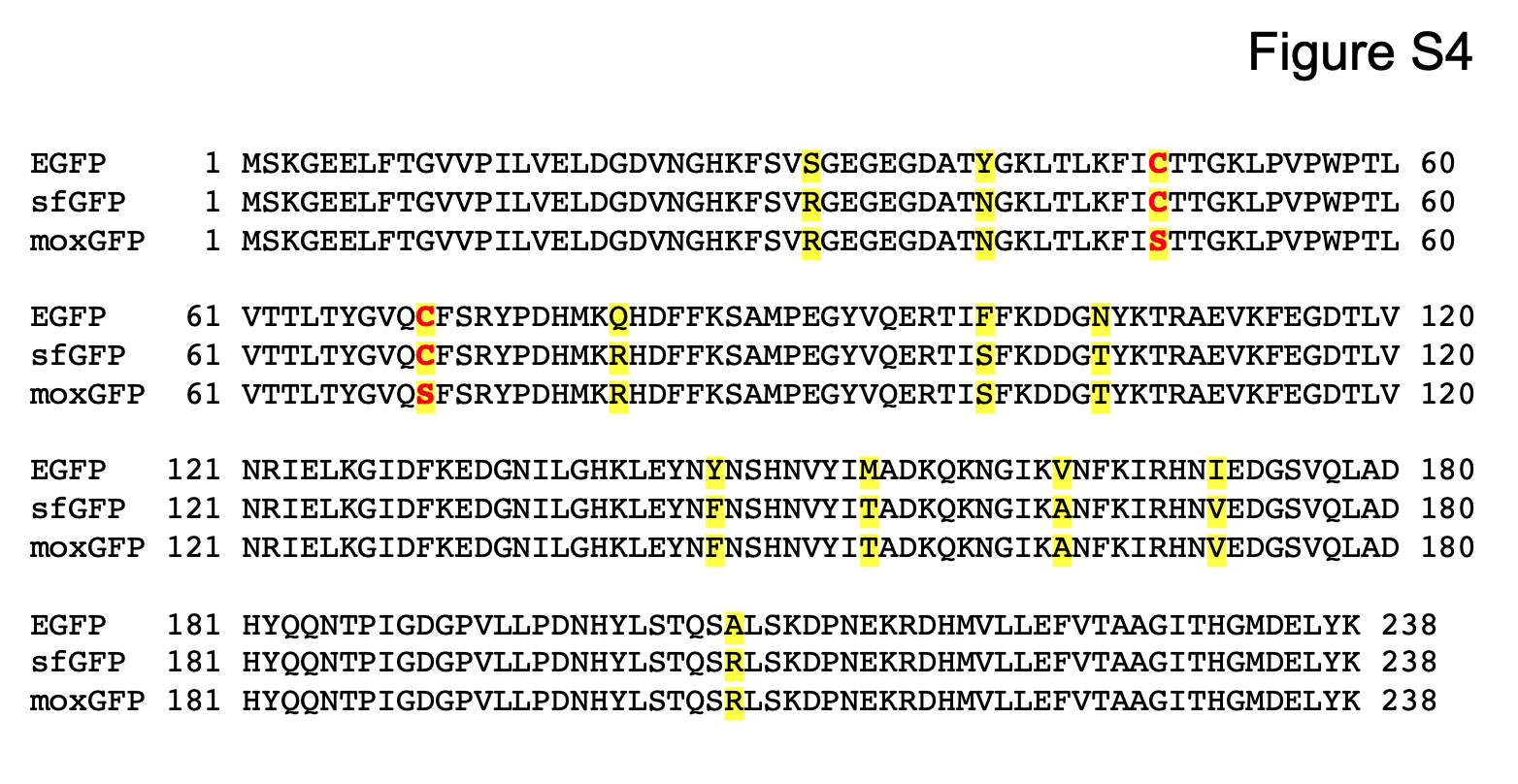


Figure S4. Amino acid sequence alignment of EGFP, sfGFP, and moxGFP. Different amino acids among the three fluorescent proteins are shown in yellow; two cysteine residues substituted in moxGFP are shown in red letters.


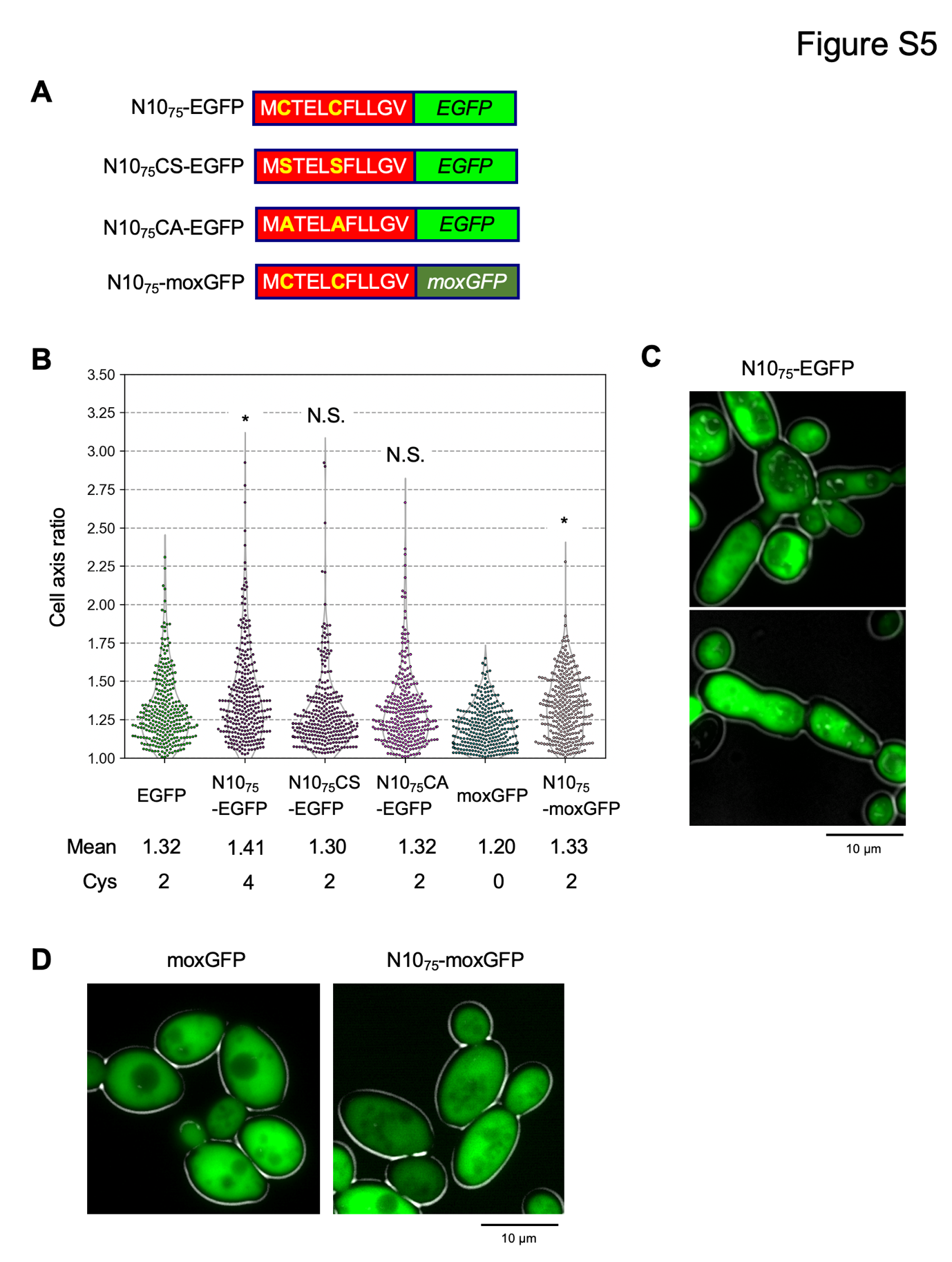


# Figure S5. The addition of cysteines to EGFP exacerbates the cell elongation phenotype

(**A**) Structure of N10-EGFP. Yellow amino acids indicate cysteine and substituted amino acids. (**B**) Swarm plot of cell axis ratio of cells overexpressing EGFP, N10_75_-EGFPs, moxGFP, and N10_75_-moxGFP. Plots were based on 300 cells from three biological replicates. *: *p* < 0.05, N.S.: *p* > 0.05, Levene’s test with Bonferroni correction in comparison to the EGFP overexpression except N10_75_-moxGFP, which is compared to the moxGFP overexpression. The mean cell axis ratio (Mean) and the cysteine content (Cys) of each protein are also shown. (**C, D**) Representative microscopic images of cells overexpressing N10-EGFP (**C**), moxGFP and N10_75_-moxGFP (**D**); for fluorescent protein images, bright-field and pseudo-color fluorescence images were merged. The cells were cultured in –Leu/Ura conditions.


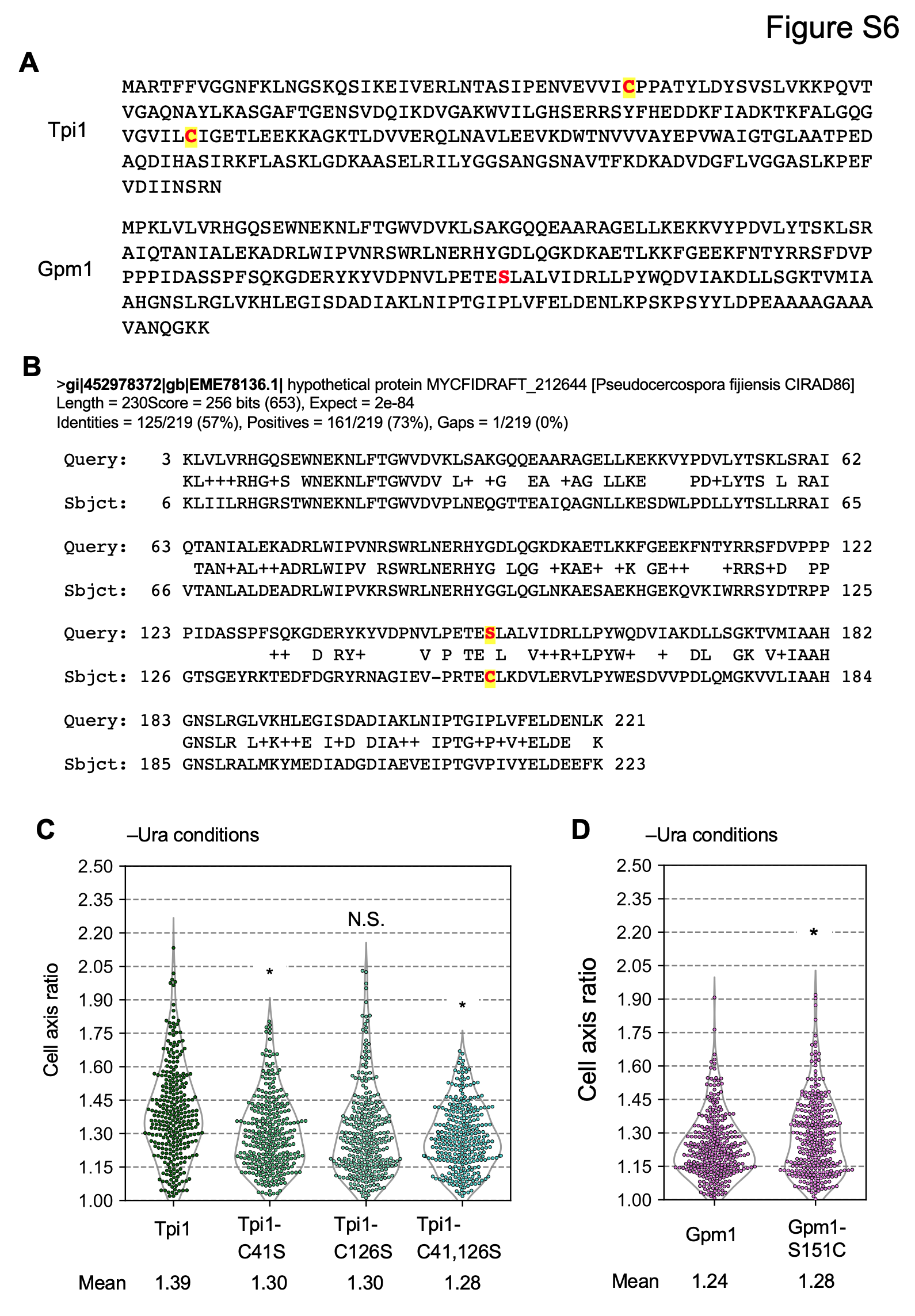


# Figure S6. Cysteine residues in the glycolytic enzymes Tpi1 and Gpm1 are associated with the cell elongation phenotype caused by their overexpression.

(**A**) Amino acid sequences of Tpi1 and Gpm1. The substituted cysteine and serine residues are colored. (**B**) Alignment of Gpm1 in *S. cerevisiae* and a hypothetical Gpm1 homolog in *Pseudocercospora fijiensis*. Amino acids corresponding to the serine substituted with the Gpm1-S151C mutant are colored. Alignments were performed by NCBI BLAST (<https://blast.ncbi.nlm.nih.gov/Blast.cgi>) (**C**) Swarm plot of cell axis ratio of cells Tpi1 and Tpi1 mutants with cysteine to serine substitutions. Plots were based on 300 cells from three biological replicates. In comparison to the wild type Tpi1 overexpression, *: *p* < 0.05, N.S.: *p* > 0.05, Levene’s test with Bonferroni correction. (**D**) Swarm plot of cell axis ratio of cells Gmp1 and Gpm1 mutants with serine 151 to cysteine substitution. Plots were based on 300 cells from three biological replicates. In comparison to the wild type Gpm1 overexpression, *: *p* < 0.05, N.S.: *p* > 0.05, Levene’s test. The mean cell axis ratio (Mean) and the cysteine content (Cys) of each protein are also shown. The cells were cultured in –Ura conditions.


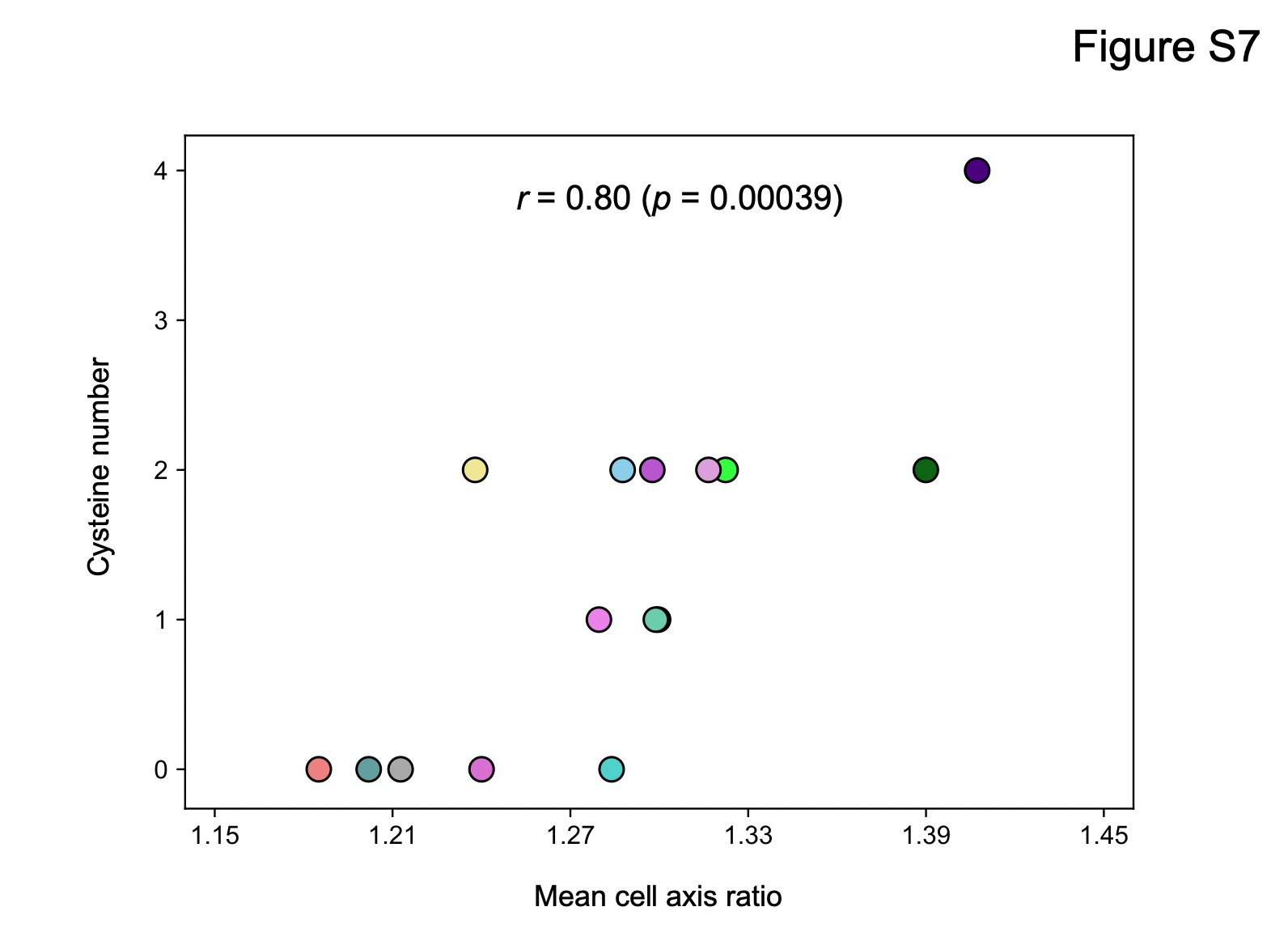


# Figure S7. Relationship between the cysteine content of the proteins investigated in this study and the cell elongation phenotype (mean cell axis ratio) when they are overexpressed.

Pearson's correlation coefficient (r) and its p-value are also shown.


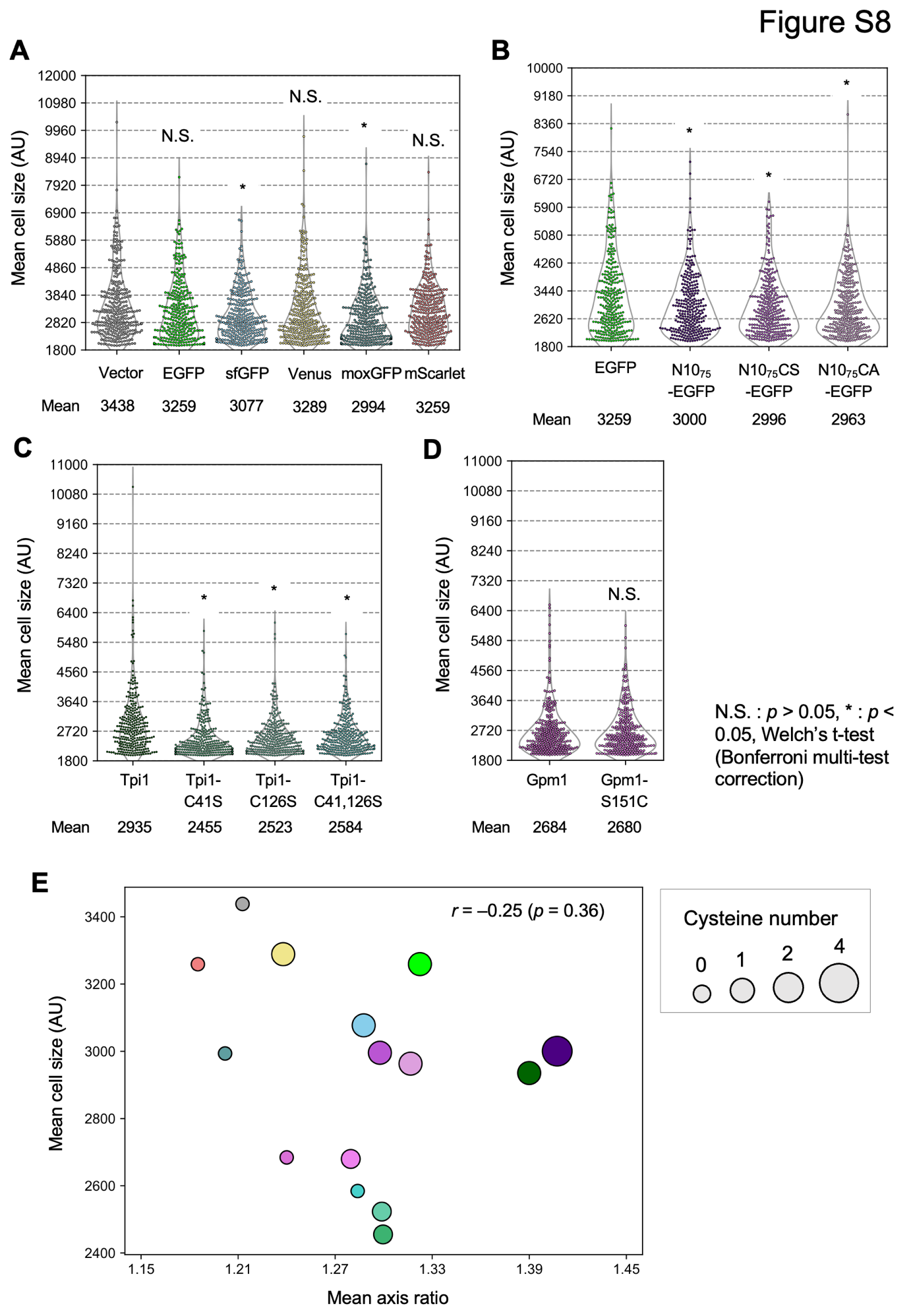


# Figure S8. Size of cells overexpressing proteins.

(**A**-**D**) Swarm plots showing the size of yeast cells overexpressing fluorescent proteins (**A**), N1075-GFP (**B**), Tpi1 (**C**), and Gpm1 (**D**) and their mutants. N.S. : p > 0.05, * : p < 0.05, Welch's t-test (Bonferroni multi-test correction). The cells were cultured under SC–Leu/Ura conditions in **A** and **B**, and under SC–Ura conditions in **C** and **D**. (**E**) Relationship between cell size and cell elongation phenotype. The cysteine content of each protein is indicated by the size of the circle. Pearson's correlation coefficient (r) and its p-value are also shown.

**
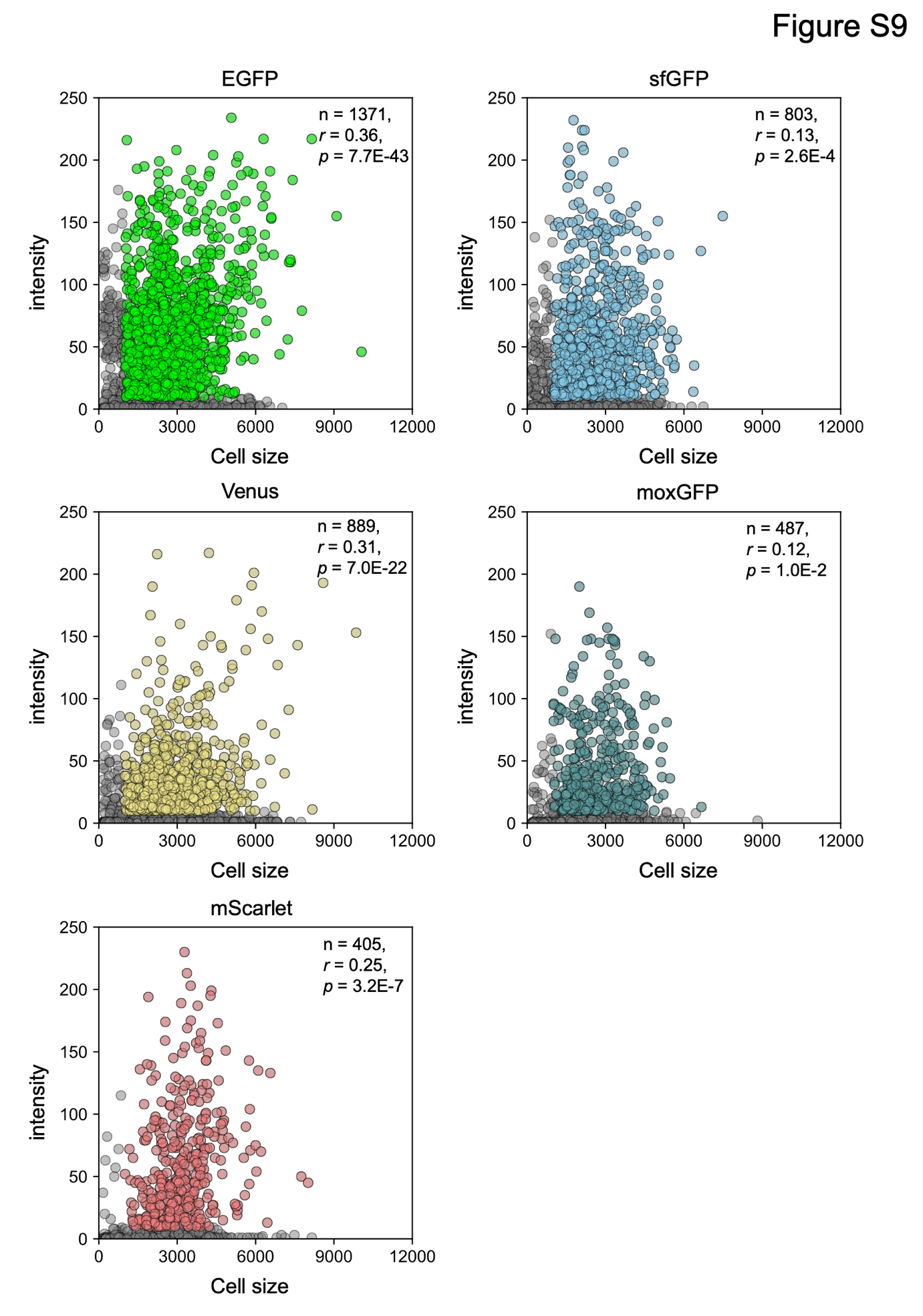
**

# Figure S9. Scatterplots for cell size and fluorescence intensity.

Cell morphology analysis was performed on all cells recognized from the microscopy images (not randomly selected). Gray dots indicate data with intensities less than or equal to 10 or cell sizes less than 1000. n: number of cells indicated in non-gray, *r*: Pearson's correlation coefficient, *p*: test for uncorrelatedness. The cells were cultured under SC–Leu/Ura conditions.

**
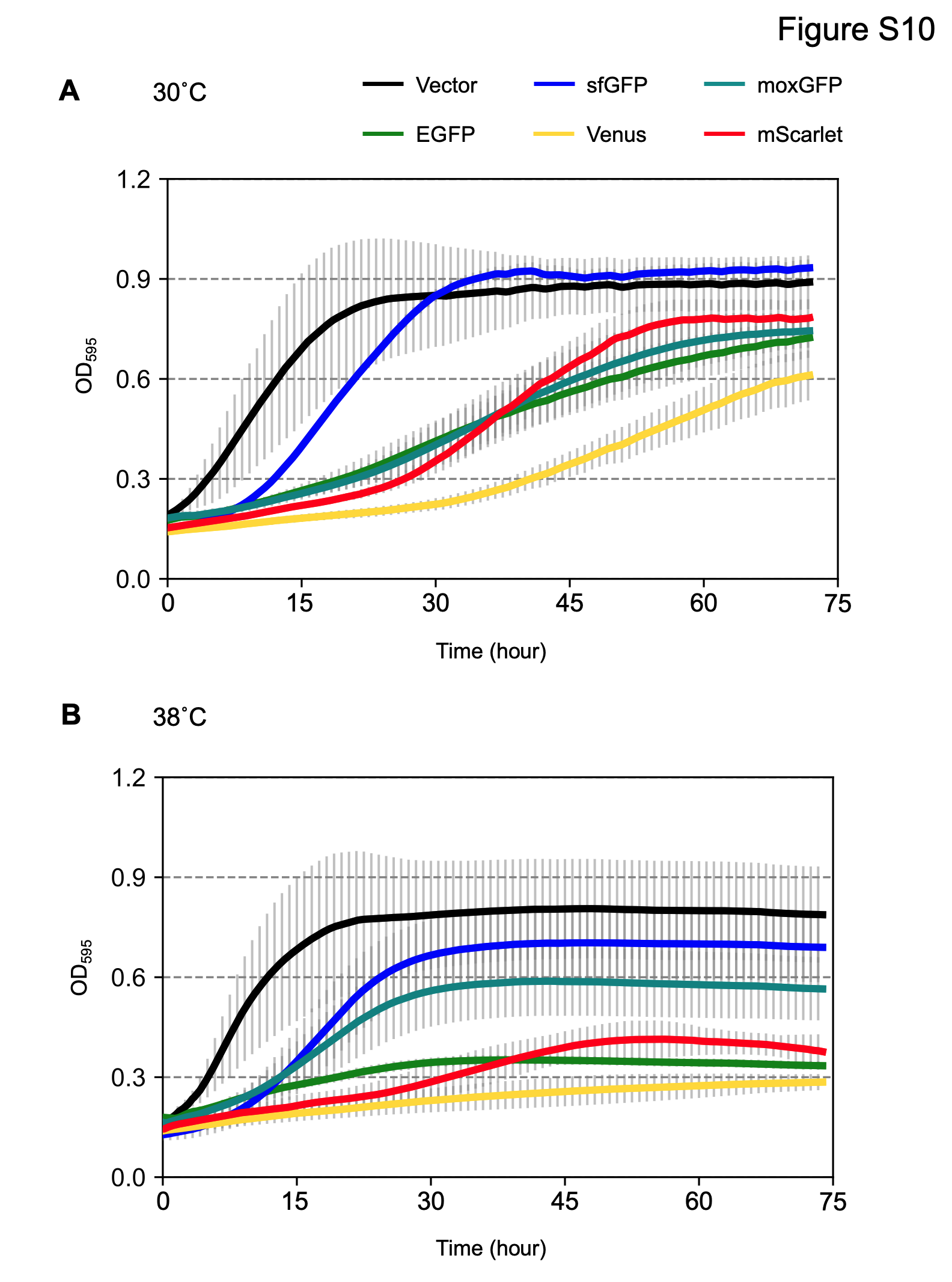
**

# Figure S10. Growth curve of cells overexpressing fluorescent proteins.

**(A** and **B)** Growth in SC–Leu/Ura medium at 30°C (**A**) and 38°C (**B**) was monitored with a microplate reader. Thick curves and thin vertical lines represent the mean and standard deviation of the eight biological replicates of turbidity measured at an optical density of 595 nm (OD_595_), respectively.

**
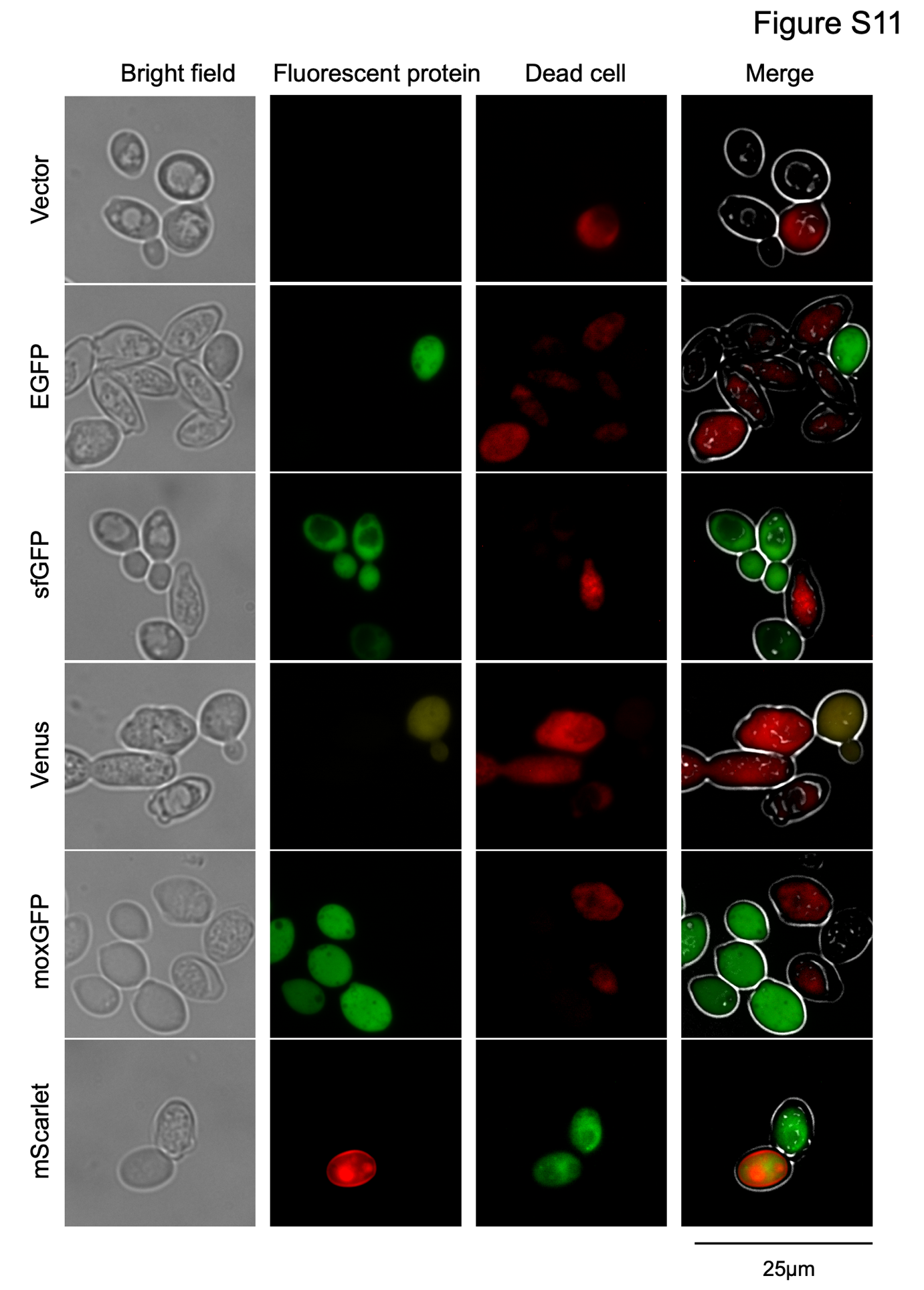
**

Figure S11. Microscopic images of cells overexpressing fluorescent proteins cultured at 38°C. Dead cells were stained with propidium iodide in EGFP, sfGFP, Venus, and moxGFP-overexpressing cells and with SYTOX green in mScarlet-overexpressing cells.

**
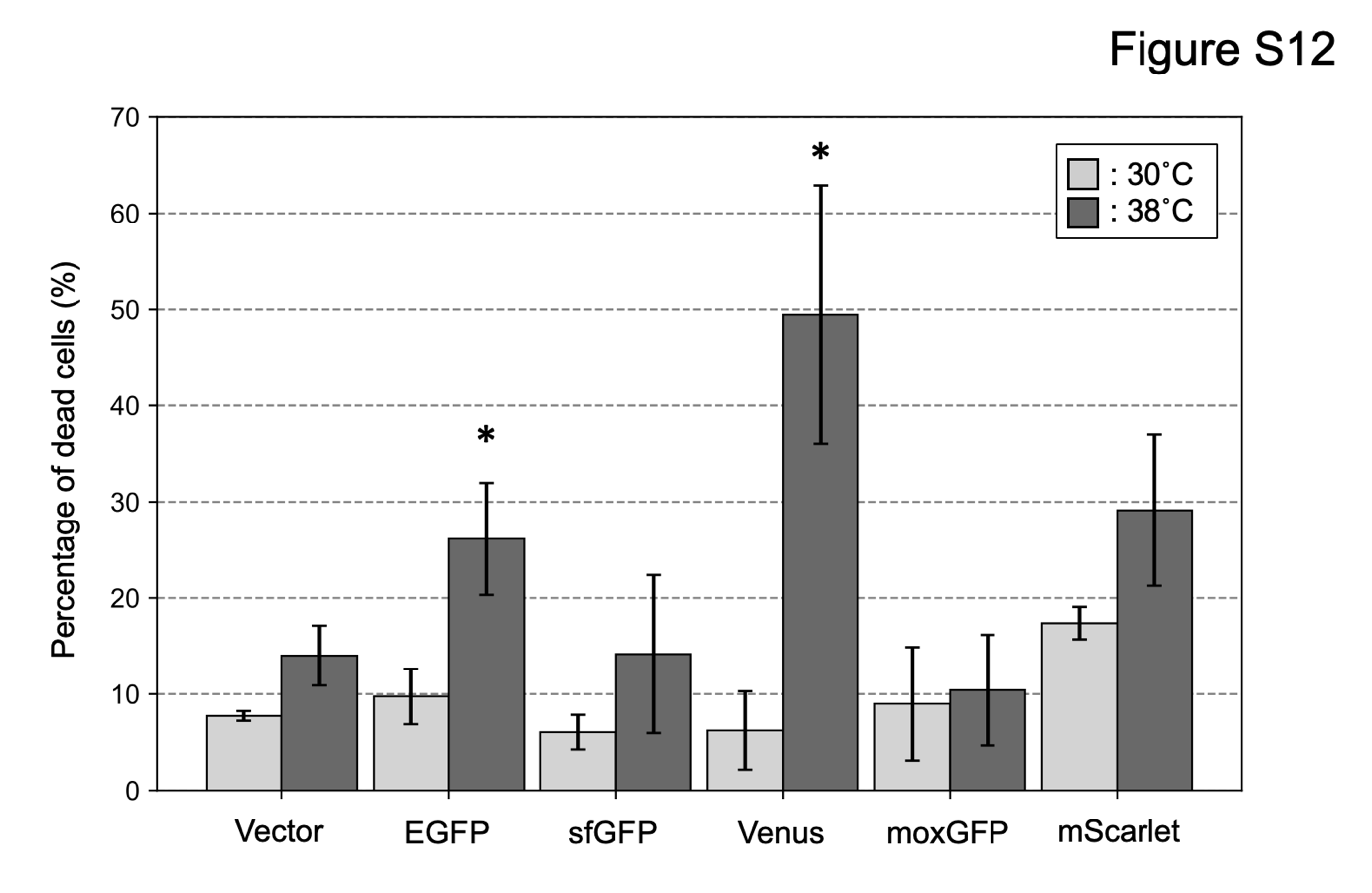
**

Figure S12. Percentage of dead cells in a cell population overexpressing fluorescent proteins. The percentage of dead cells (i.e. cells stained with propidium iodide or SYTOX green) was examined for at least 300 cells stained as shown in Figure 11. The bars and error bars represent the mean and standard deviation in three replicates. In comparison between 30°C and 38°C, *: *p* < 0.05, Welch’s test.

**
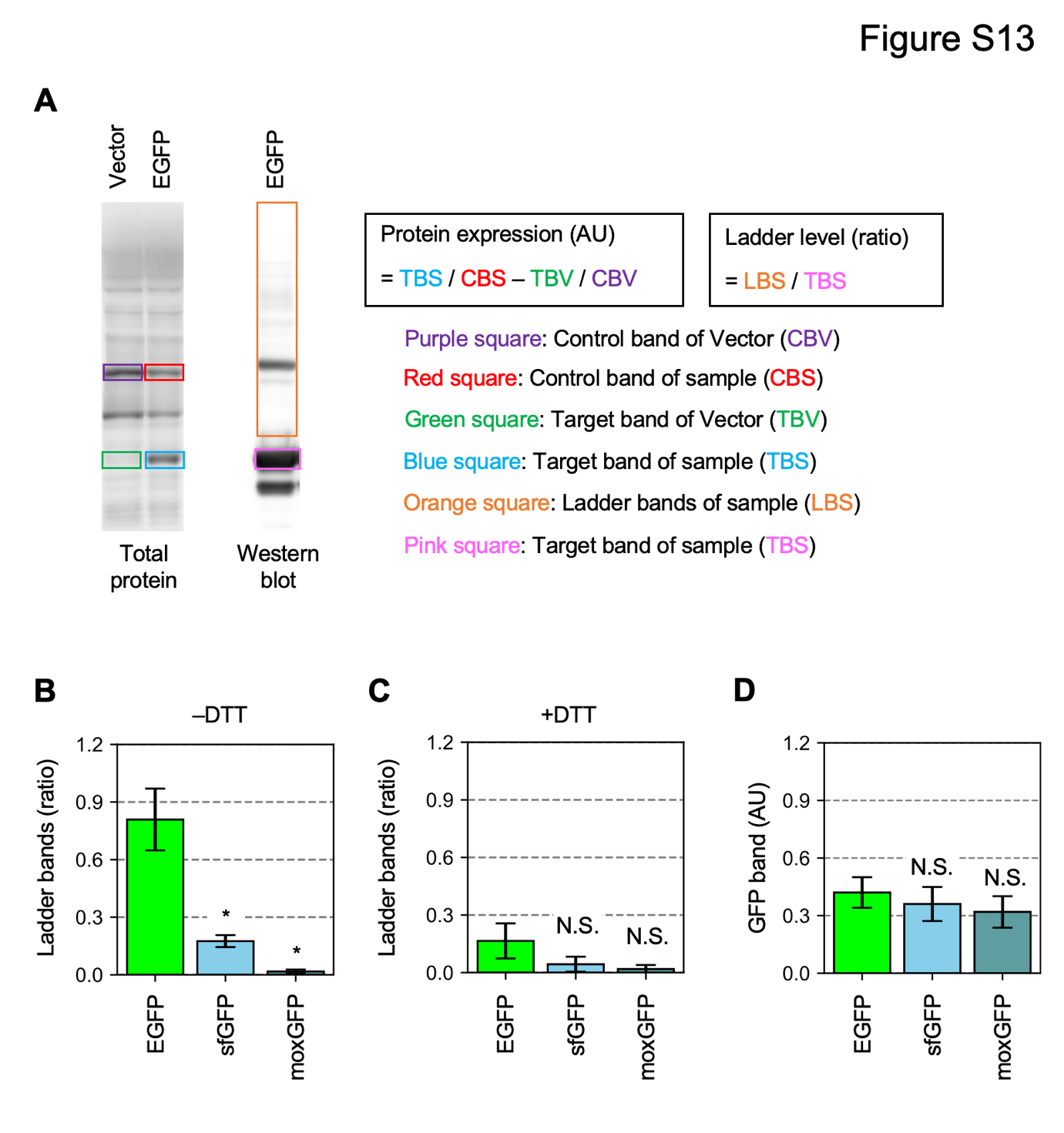
**

# Figure S13. Quantification of overexpressed proteins and aggregation bands.

**(A)** Measurement and calculation of the amount of overexpressed proteins and aggregation bands. The extracted cellular proteins were stained with a fluorescent dye and separated by SDS-PAGE.﻿ GFP was detected by Western blotting using an α-GFP antibody. Protein expression levels (AU) were calculated by normalizing the overexpression of target proteins (TBS), as estimated from band intensity and molecular weight on the gel, using control bands (CBS, CBV) and then comparing them with the expression of the corresponding vector control (TBV). The ladder level is the ratio of the high-molecular-weight band (LBS) above the target protein normalized by the band of the target protein (TBS) detected by Western blotting. **(B and C)** Quantification of GFP aggregation bands in DTT-untreated (–DTT, **A**) and -treated (+DTT, **B**) samples. **(D)** Quantification of GFP bands in DTT-untreated samples. In comparison with EGFP, *: *p* < 0.05, N.S.: *p* > 0.05, Welch’s test with Bonferroni correction. Cells cultured in –Ura were used.


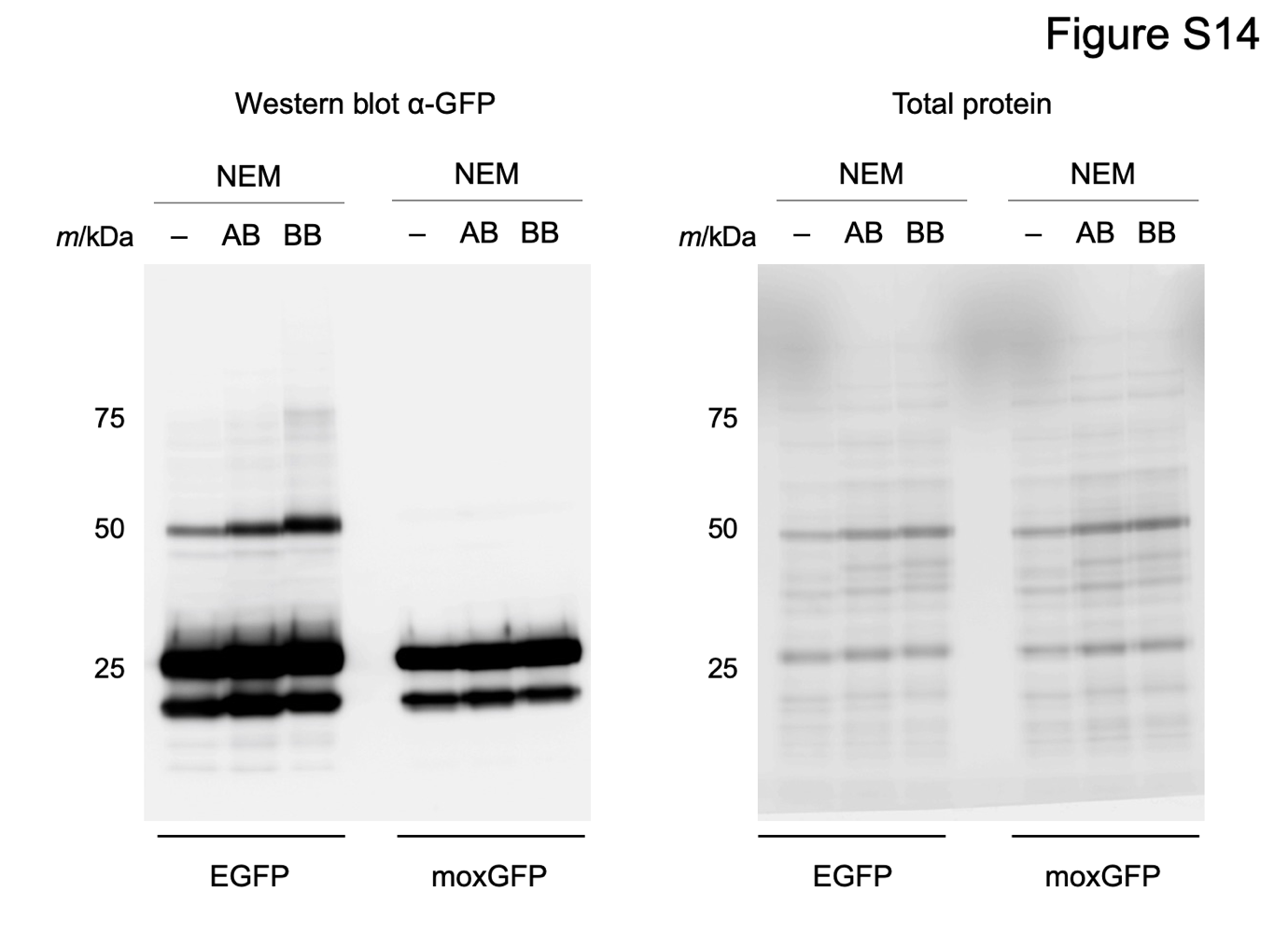


Figure S14. Evidence that disulfide bonding of EGFP occurs intracellularly, not during protein extraction. Western blotting and total protein images of proteins extracted from cells overexpressing EGFP and moxGFP. Samples are treated with 20mM N-ethylmaleimide (NEM) before boiling (BB), or after boiling (AB). Note that cells are destroyed by boiling.

**
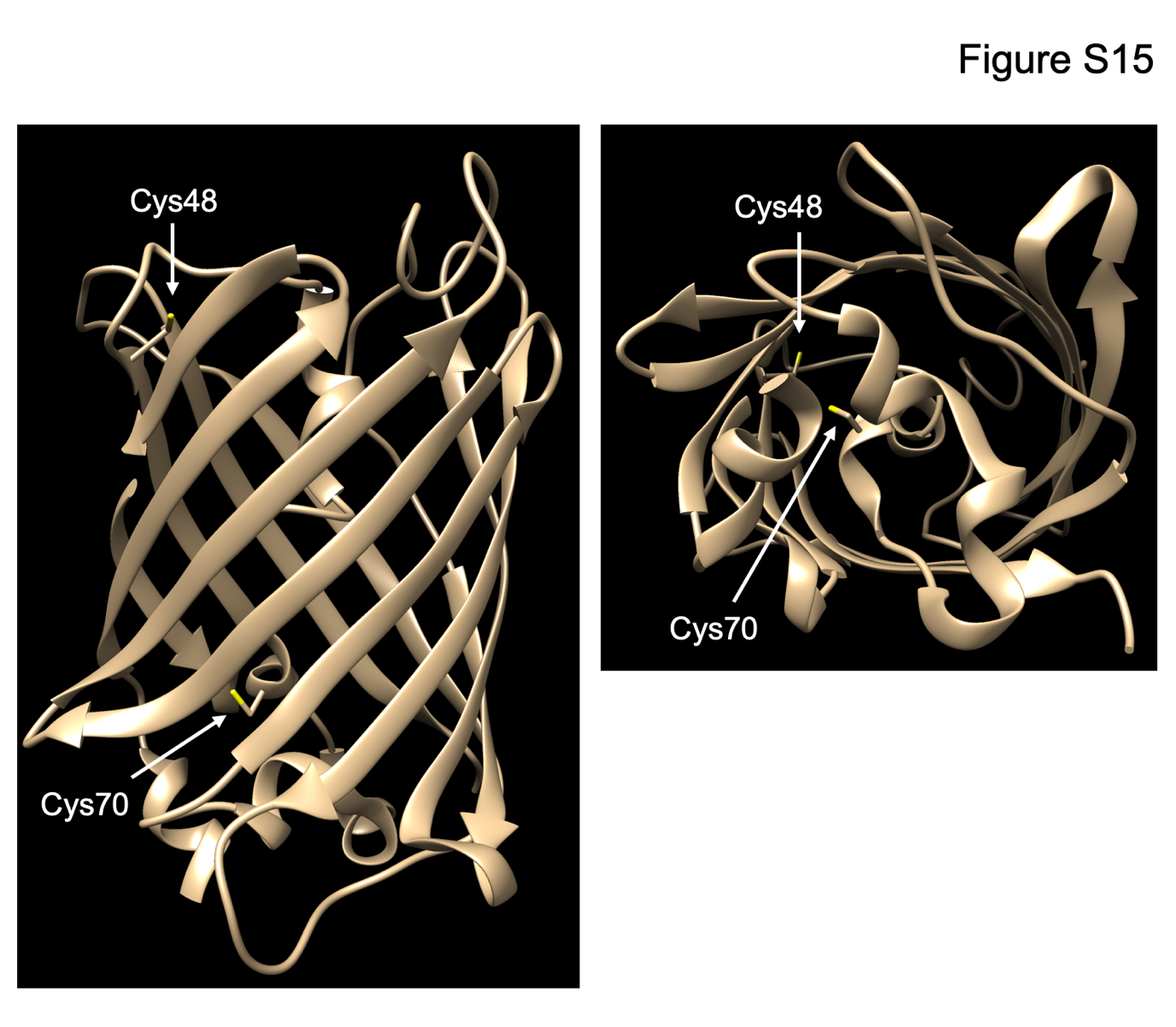
**

# Figure S15. The thiol groups of the two cysteines of EGFP are located inside the structure.

The 3D structure model of EGFP (PDB, 2Y0G) from two perspectives and the two cysteines are represented.


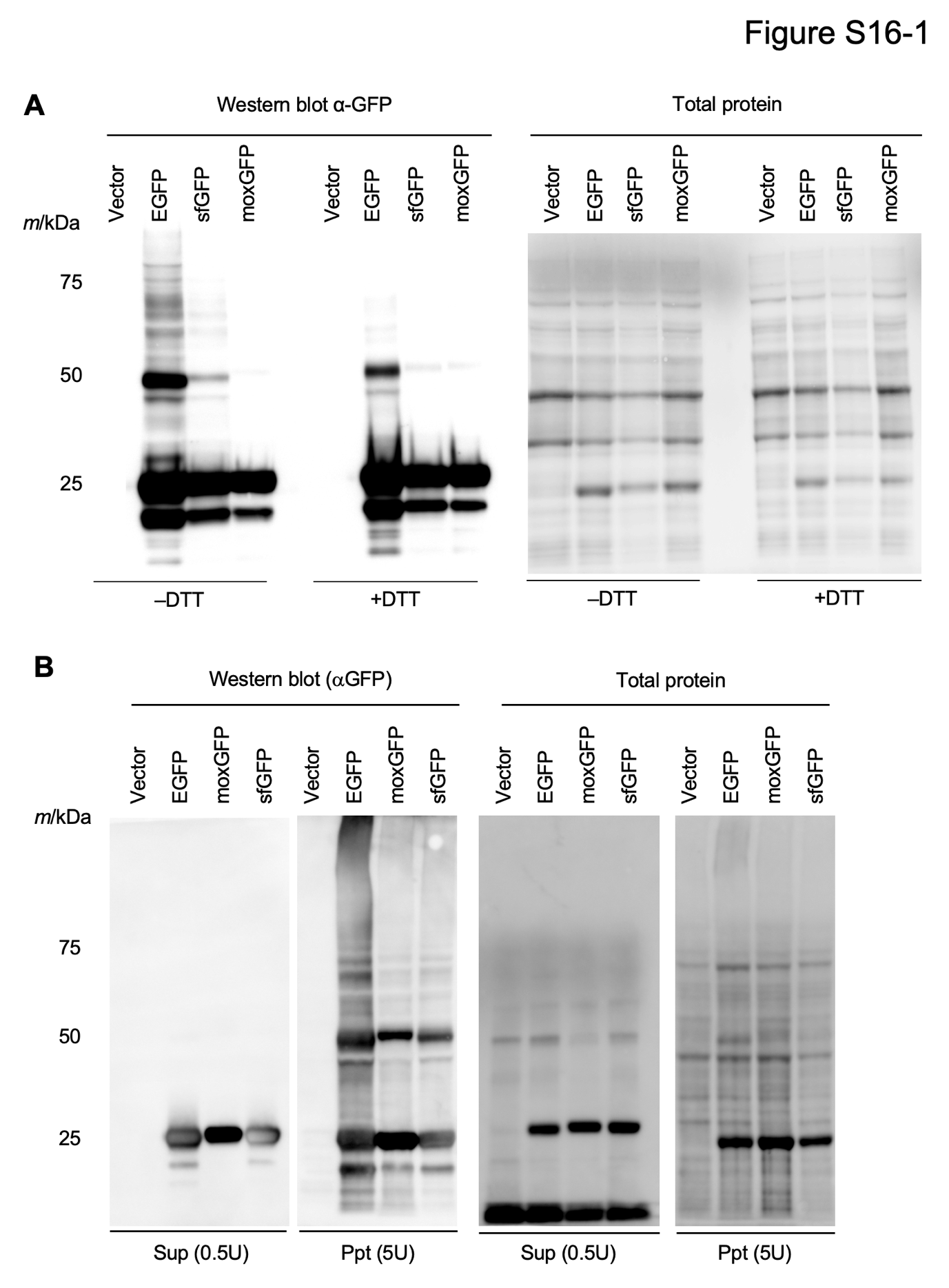


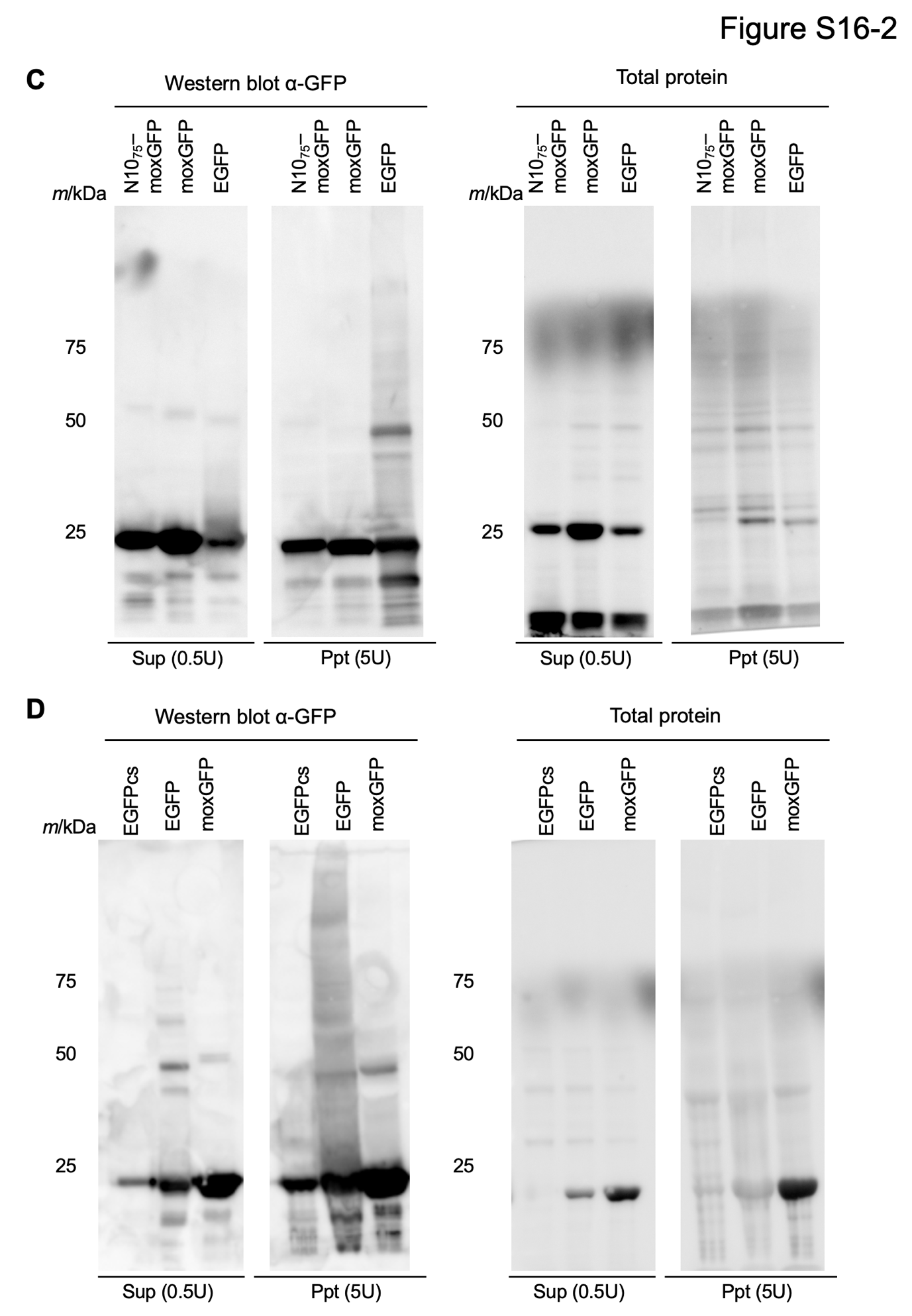


# Figure S16. Overexpression of EGFP causes protein aggregation via the S-S bond.

**(A-D)** Western blotting images with the total protein images used in Figure 5.


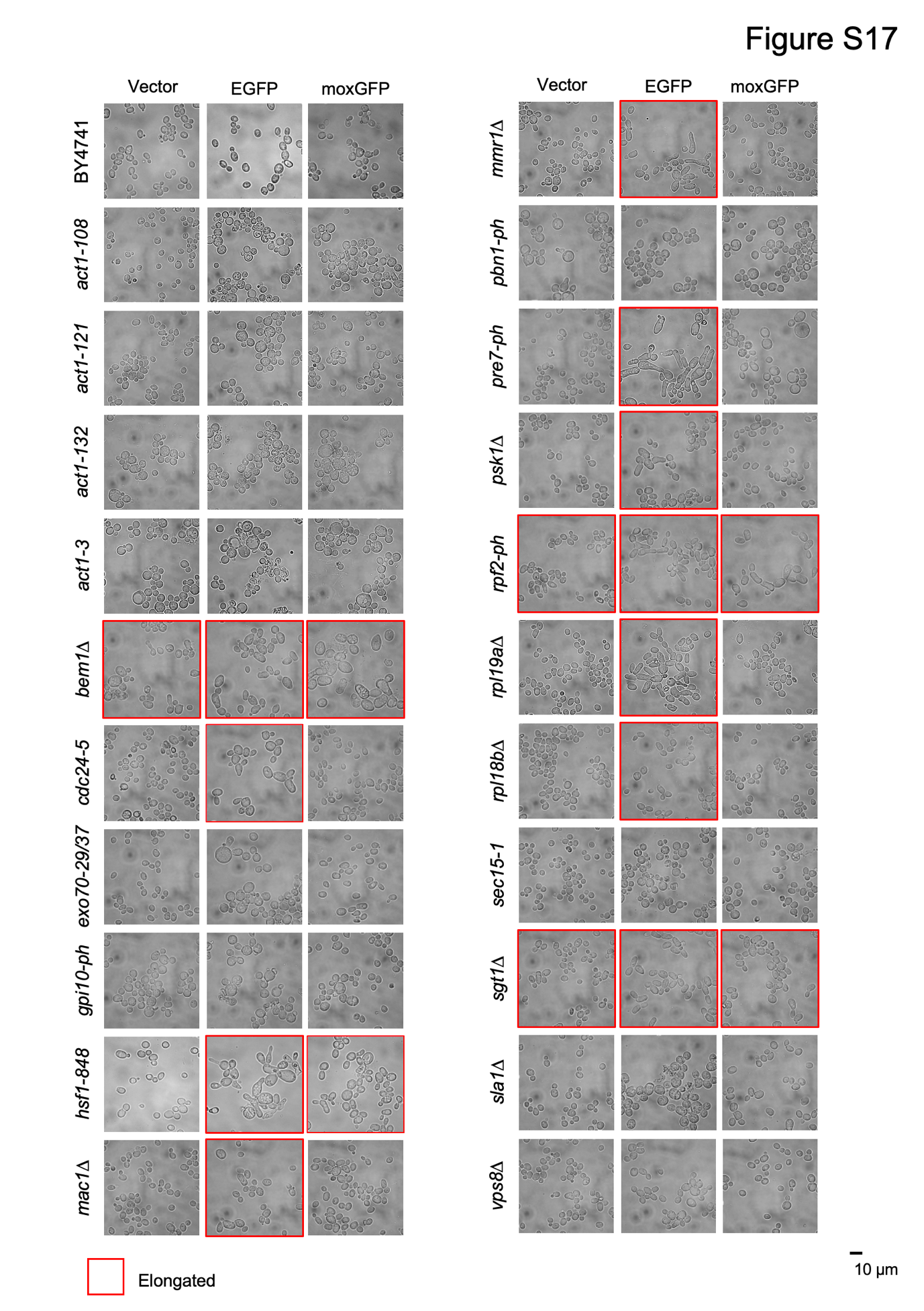


# Figure S17. Microscopic images of cells overexpressing EGFP and moxGFP, as well as the vector control cells.

EGFP and moxGFP were overexpressed in indicated mutant cells and the cell images were taken. The cells were cultured in –Leu/Ura conditions at 30˚C. Strains with visually confirmed cell elongation are boxed in red.

**
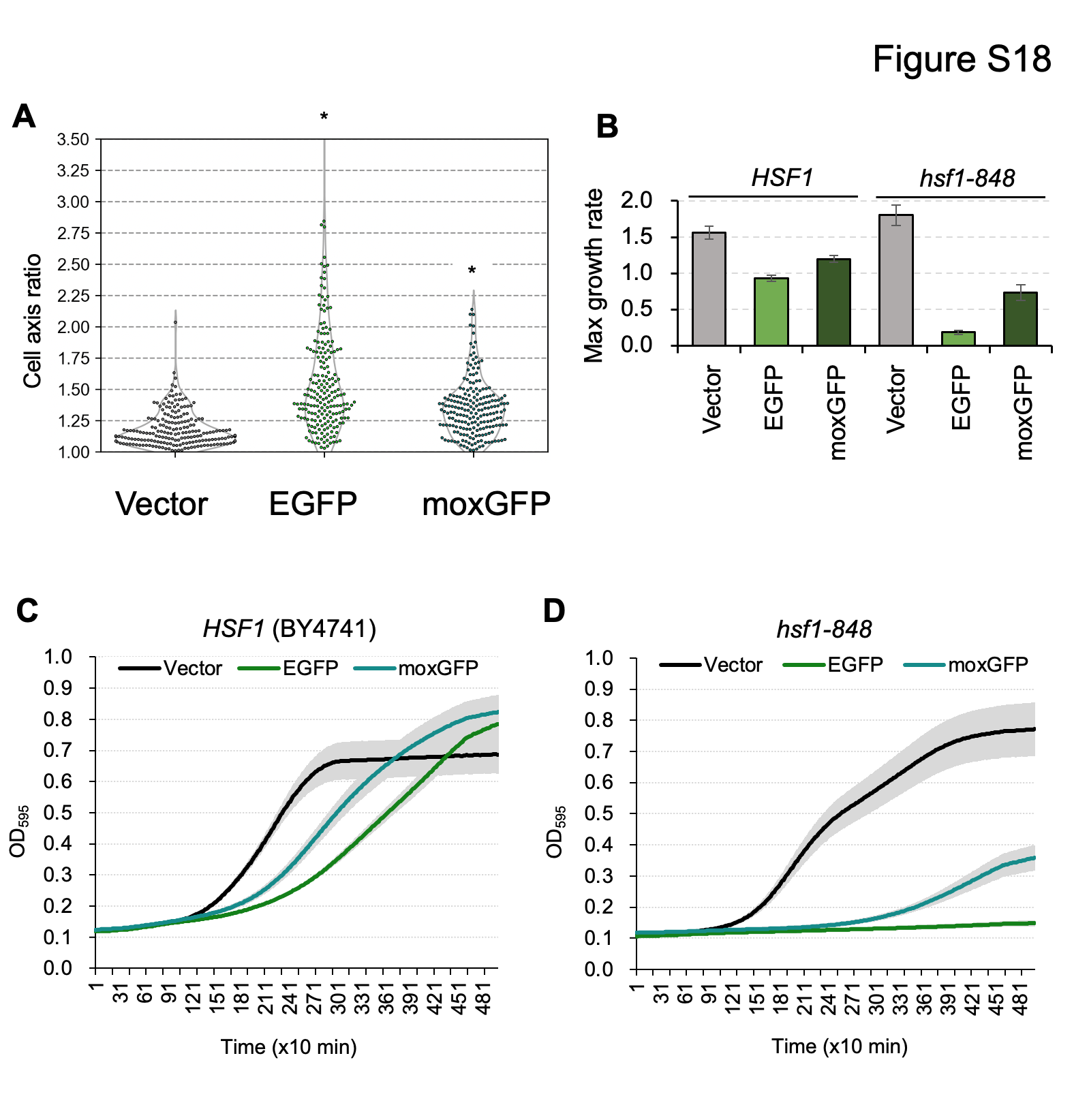
**

# Figure S18. Effects of EGFP and moxGFP overexpression on the hsf1-848 mutation

(**A**) Swarm plot of cell axis ratio of cells overexpressing the indicated fluorescent proteins and the vector control. Plots were based on **300** cells from five images. In comparison to the vector control, *: *p* < 0.05, N.S.: *p* > 0.05, Levene’s test. The cells were cultured in –Leu/Ura conditions. (**B**) Max growth rate of wild-type (*HSF1*, BY4741) and *HSF1* temperature-sensitive mutant (*hsf1-848*) strains upon overexpression of EGFP and moxGFP. (**C** and **D**) Growth curve of cells overexpressing EGFP and moxGFP in the wild type (*HSF1*, BY4741) and the *HSF1* temperature sensitive mutant (*hsf1-848*) strains. Growth in SC-Leu/Ura medium at 30°C was monitored with a microplate reader. Thick curves and gray zones represent the mean and standard deviation of the eight biological replicates of turbidity measured at an optical density of 595 nm (OD_595_), respectively.

#
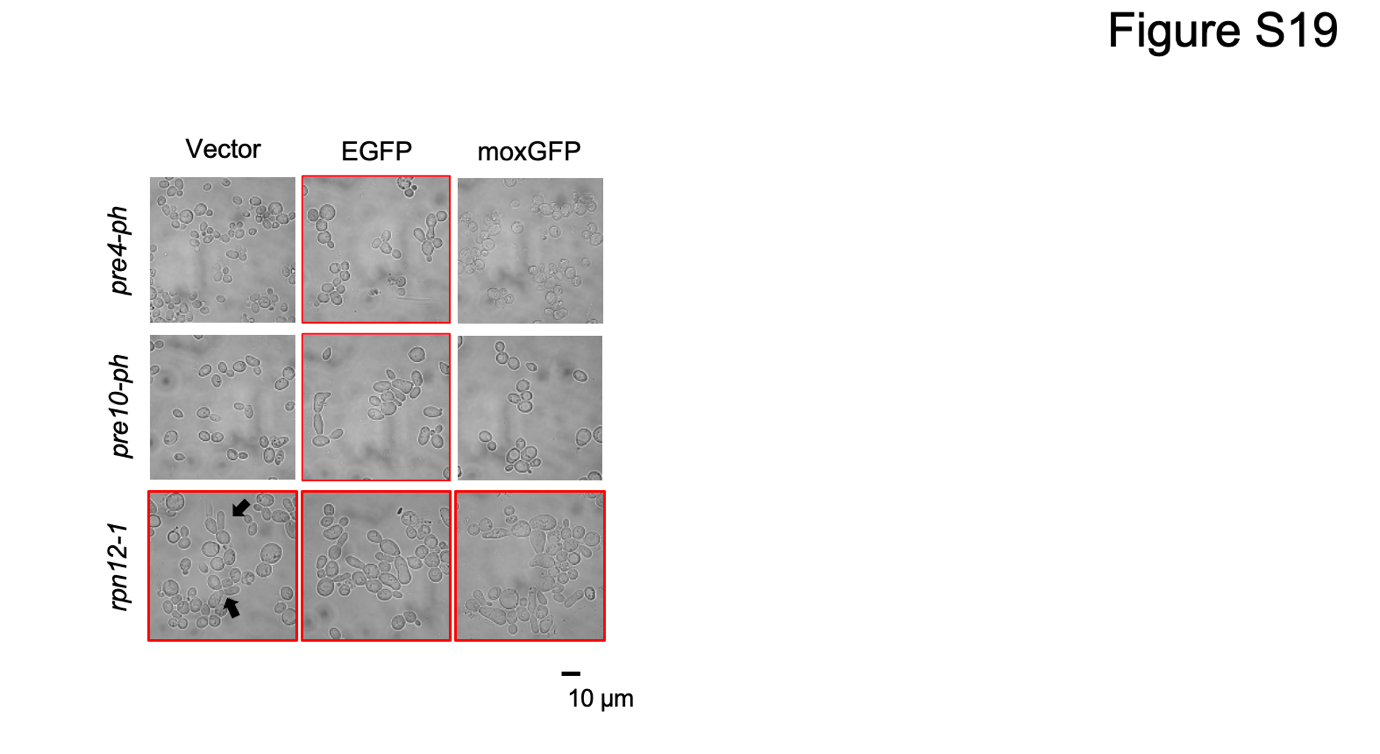


Figure S19. Microscopic images of mutant cells overexpressing EGFP and moxGFP, as well as the vector control cells.

EGFP and moxGFP were overexpressed in indicated mutant cells and the cell images were taken. The cells were cultured in –Leu/Ura conditions at 30˚C. The cells were cultured in –Leu/Ura conditions. Strains with visually confirmed cell elongation are boxed in red.


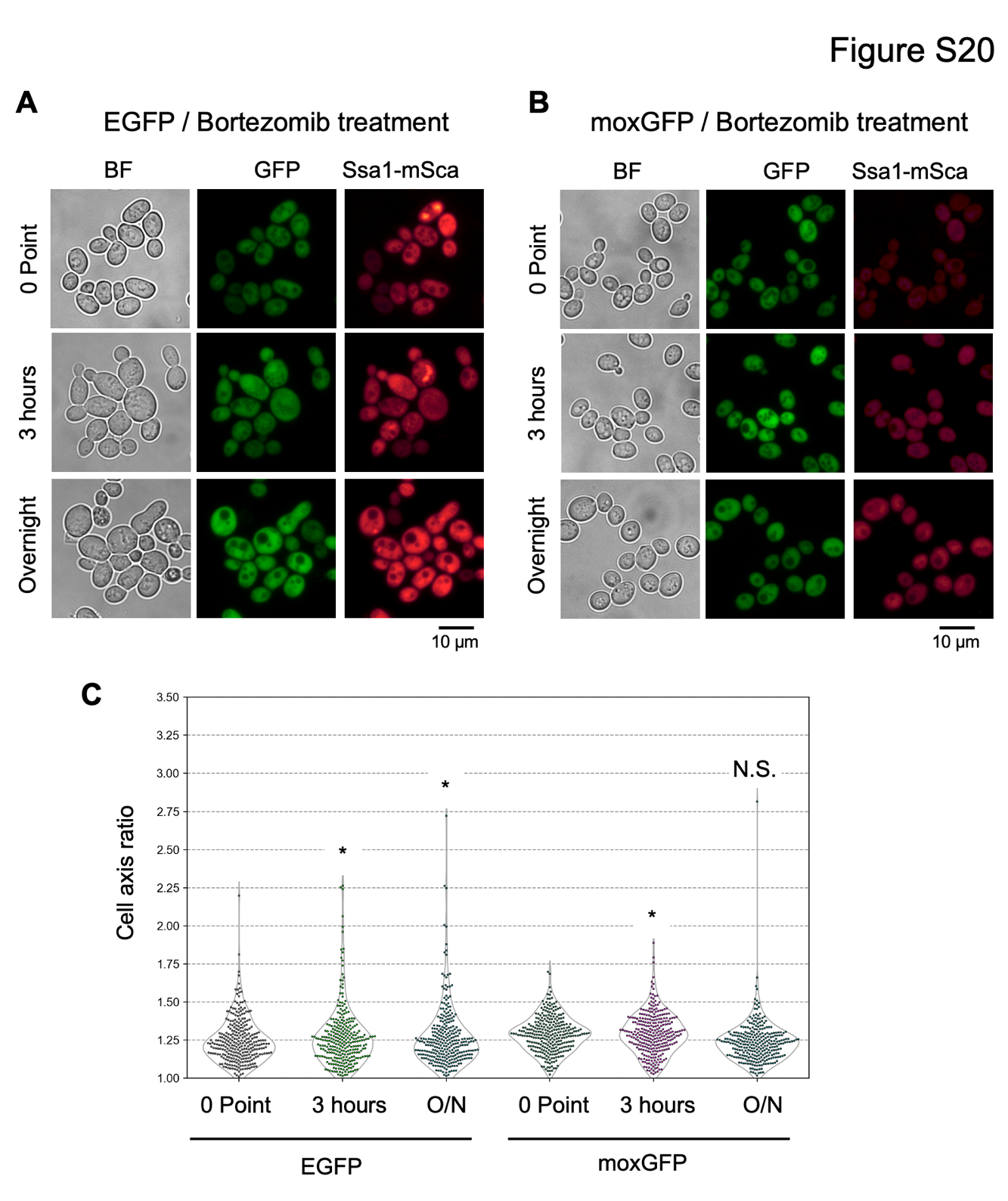


Figure S20. Bortezomib treatment of overexpressing cells

(**A** and **B**) BY4741 cells overexpressing EGFP and moxGFP along with Ssa1-mScarlet-I were cultured in SC-Leu/Ura until OD_660_ of 1.0., bortezomib (final concentration 100µM) was added, and fluorescence microscopy imaging was performed immediately (0 point), 3 hours, and overnight. Subcellular localization of Ssa1-mSca was also analyzed. Ssa1-mScaI: red fluorescent image of Ssa1-mScarlet-I, GFP: green fluorescent image of GFP variants. (**C)** Swarm plot of cell axis ratio of cells overexpressing the indicated fluorescent proteins and the vector control. Plots were based on 300 cells from five images. In comparison to the 0 point, *: *p* < 0.05, N.S.: *p* > 0.05, Levene’s test.


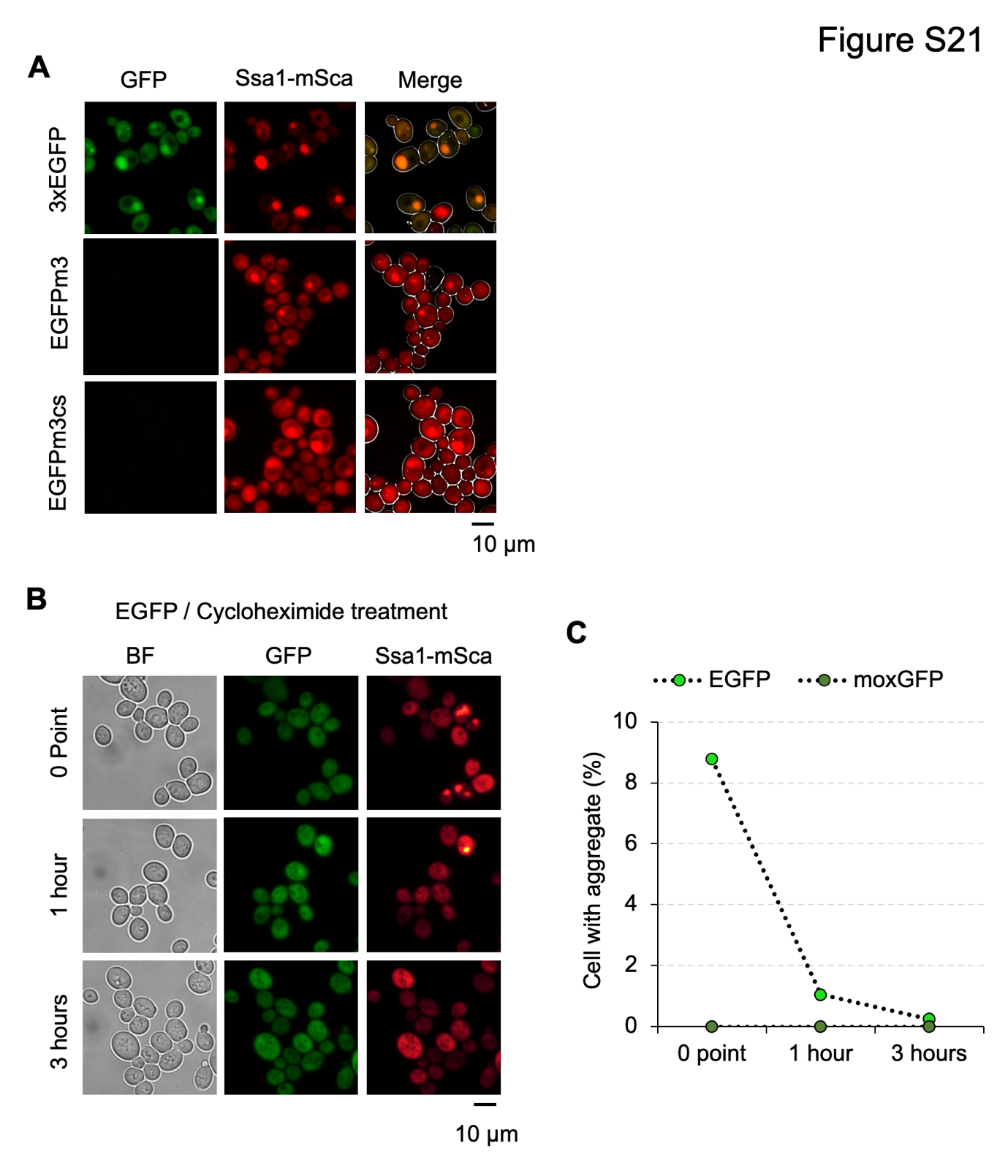


# Figure S21. Behavior of Ssa1 aggregates in overexpressing cells

(**A** and **B**) BY4741 cells overexpressing 3xEGFP, EGFPm3, and EGFPm3cs along with Ssa1-mScarlet-I were cultured in SC-Leu/Ura until OD_660_ of 1.0. Ssa1-mScaI: red fluorescent image of Ssa1-mScarlet-I, GFP: green fluorescent image of GFP variants, Merge: superimposed image of both fluorescence and cell outlines obtained from brightfield image. Note that EGFm3 and EGFPm3cs tend to misfold, so there is no GFP fluorescence. (**B**) Cycloheximide treatment of overexpressing cells. BY4741 cells overexpressing EGFP and moxGFP along with Ssa1-mScarlet-I were cultured in SC-Leu/Ura until OD_660_ of 1.0, cycloheximide (final concentration 250µg/ml) was added, and fluorescence microscopy imaging was performed immediately (0 point), 3 hours, and overnight. Ssa1-mScaI: red fluorescent image of Ssa1-mScarlet-I, GFP: green fluorescent image of GFP variants. (**C**) Quantitative measurement of Ssa1-mScarlet-I aggregates. The aggregates observed in more than 300 cells cultured by method B were quantified by visually and expressed as a percentage.


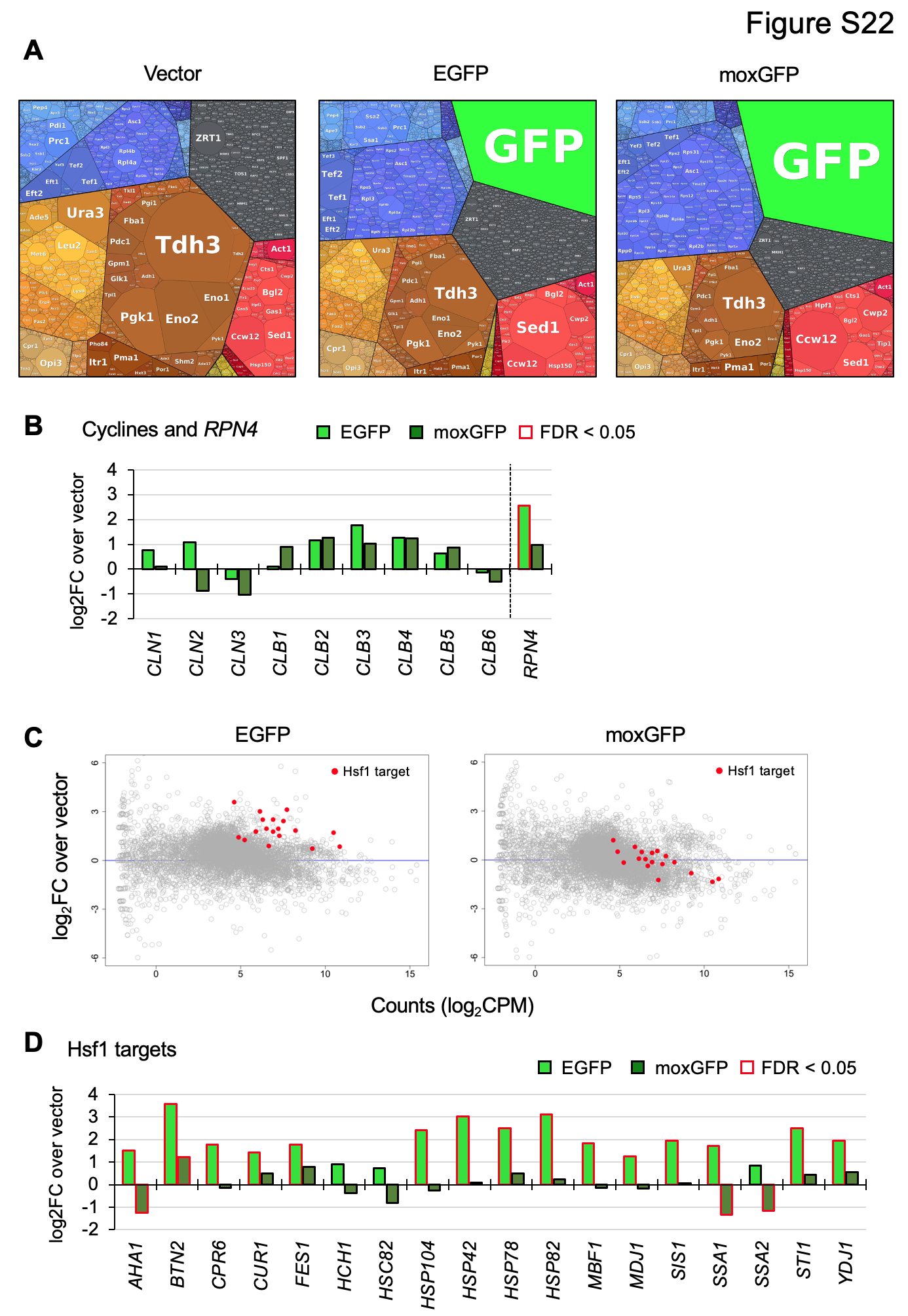


# Figure S22. Transcriptome analysis of EGFP and moxGFP overexpressing cells

(**A**) Visualization of RNAseq data using Proteomap. RNAseq read data was visualized by the Proteomap (<https://bionic-vis.biologie.uni-greifswald.de/>) with a custom treemap template.

(**B**) Cyclin genes *RPN4* were extracted and plotted as a bar graph from the same data in **C**. Data with significant false discovery rate (FDR < 0.05) are indicated by red boxes. **(C**) Transcriptional profiles of EGFP and moxGFP overexpression strains obtained by RNAseq analysis. Each gray circle represents the change in the read count of the respective gene in the overexpressing strain over the vector (log_2_ fold change, FC), and the average read count of the overexpressing strain and the vector (log_2_ counts per million reads, CPM). The red filled circles indicate genes known to be targets of Hsf1. **(D)** The targets of Hsf1 were extracted and plotted as a bar graph from the same data in **C**. Data with significant false discovery rate (FDR < 0.05) are indicated by red boxes.
